# Responses of birds and mammals to long-established wind farms in India

**DOI:** 10.1038/s41598-022-05159-1

**Published:** 2022-01-25

**Authors:** Honnavalli N. Kumara, S. Babu, G. Babu Rao, Santanu Mahato, Malyasri Bhattacharya, Nitin Venkatesh Ranga Rao, D. Tamiliniyan, Harif Parengal, D. Deepak, Athira Balakrishnan, Mahesh Bilaskar

**Affiliations:** 1 Sálim Ali Centre for Ornithology and Natural History, Anaikatty, Coimbatore, Tamil Nadu 641108 India; 2 Manipal Academy of Higher Education, Madhav Nagar, Manipal, Karnataka 576104 India; 3 Biopsychology Laboratory, Institution of Excellence, University of Mysore, Mysuru, Karnataka 570006 India; 4 Wildlife Institute of India, Chandrabani, Dehradun, Uttarakhand 248001 India; 5 Bharathiar University, Coimbatore, Tamil Nadu India; 6 National Institute of Advanced Studies, Indian Institute of Science Campus, Bangalore, Karnataka 560012 India; 7 Department of Environmental Sciences, Savitribai Phule Pune University, Ganeshkhind Road, Pune, 411007 Maharashtra India

**Keywords:** Ecology, Zoology, Ecology, Environmental sciences

## Abstract

Wind turbines have been recognised as an alternative and clean-energy source with a low environmental impact. The selection of sites for wind-farm often creates serious conservation concerns on biodiversity. Wind turbines have become a serious threat to migratory birds as they collide with the turbine blades in some regions across the globe, while the impact on terrestrial mammals is relatively less explored. In this context, we assessed the responses of birds and mammals to the wind turbines in central Karnataka, India from January 2016 to May 2018 using carcass searches to quantify animal collisions (i.e., birds and bats), fixed radius point count for bird population parameters, and an occupancy framework for assessing the factor that determines the spatial occurrence of terrestrial mammals. The mean annual animal fatality rate per wind turbine was 0.26/year. Species richness, abundance, and unique species of birds were relatively higher in control sites over wind turbine sites. Species and functional compositions of birds in control sites were different from wind turbine sites, explaining the varied patterns of bird assemblages of different feeding guilds. Blackbuck, Chinkara, Golden Jackal, and Jungle Cat were less likely to occupy sites with a high number of wind turbines. The study indicates that certain bird and mammal species avoided wind turbine-dominated sites, affecting their distribution pattern. This is of concern to the management of the forested areas with wind turbines. We raised conservation issues and mitigating measures to overcome the negative effects of wind turbines on animals.

## Introduction

The use and demand for energy have led to a high augmentation of non-renewable energy sources like oil, natural gas, coal, and hydrocarbons which attracted global attention due to their negative impact on terrestrial ecosystems and wildlife^1–5^. The potential negative impact of energy development can result in habitat loss and fragmentation. Meanwhile, energy sources such as hydropower, wind, and solar energy, are being considered as alternative and clean energy sources to meet the growing demand for energy in the constraint of conventional energy sources^6–9^. Developing hydropower, wind, and solar energy is encouraged as dams, turbines, and solar panels require no fossil raw materials, and it is also believed that it does not pollute the environment^2,8^.

The process of tapping conventional energy sources such as hydropower, wind farms, and solar energy, has a number of severe environmental consequences. The conversion of the land to hydropower developments alters hydrology dynamics, water quality, and greenhouse gas emissions^10^. Wind resources have a lower environmental impact when compared to hydel projects^11–16^ of generating the electricity from water, however, they have been shown to be harmful to wildlife due to mortality of bats and birds due to collisions with wind turbines^17–22^. The fatality of animals was expected to be higher if the area chosen for the wind farm is rich in wildlife or falls in the migratory path of birds^23^. The fatalities due to direct collision with the rotor blades of a wind turbine, the displacement or avoidance of animals due to the construction process and the noise generated by the wind turbines, the persistence of disturbance, and habitat loss caused by the construction of wind farms and their associated infrastructure are all considered to have a negative impact on wildlife^23^.

Globally, India stands 4th position in harvesting wind energy, with an installed capacity of 37,744 MW by March 2020^24^, which, indicates the persistent efforts towards shifting to wind energy. Despite the increasing expansion of global wind energy, the impact of wind farms on terrestrial mammals is highly limited e.g., ungulates: pronghorn *Antilocapra americana*, roe deer *Capreolus capreolus*, and rocky mountain elk *Cervus elaphus*, and rodents: California ground squirrels *Spermophilus beecheyi*, and European hamster *Cricetus cricetus*^25–29^. Similarly, even in India, where the country is rich in wildlife with diverse habitats, but an understanding of the wind turbines on animals is less explored, e.g., an estimate of the animal fatality rate due to collision with wind turbines in Kutch in Gujarat and Davanagere in Karnataka^30–32^, and some parts of the northern Western Ghats of Maharashtra^33^. If wind farms are in the middle of prime wildlife habitat or on the migratory path of birds^34^, understanding the risk of animal collision or the response of animals to a wind turbine is crucial to manage or mitigate the problem, or to decide the future establishment of such farms. We investigated the current fatality rate of birds due to collision with wind turbines, the response of the diversity and composition of birds, and the occupancy pattern of terrestrial mammals in the established wind farm.

## Materials and methods

### Study site

**Figure 1:**
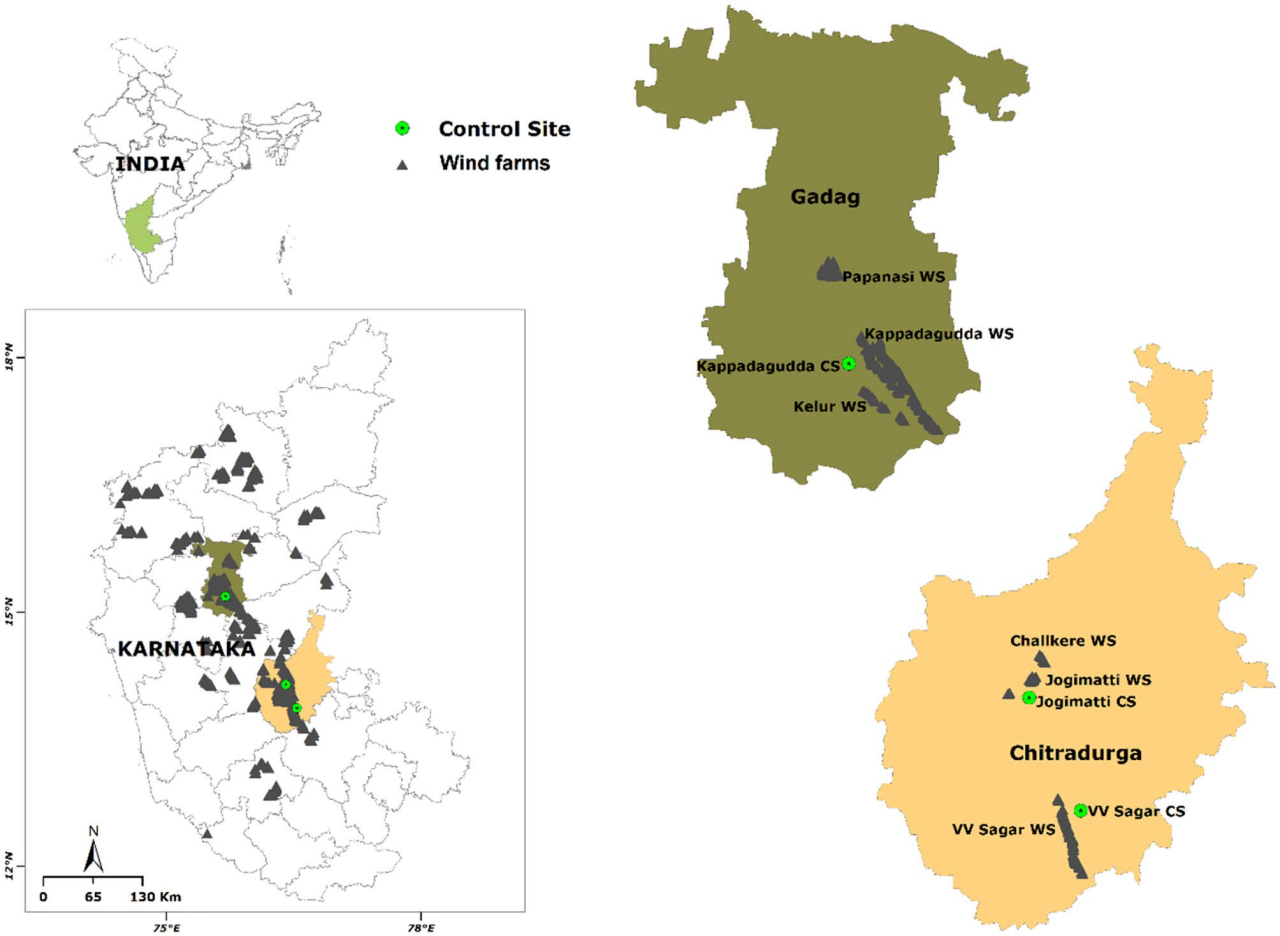
The select wind farms and control sites studied for animal fatality rate, and bird diversity and mammal distribution in Karnataka. The map is prepared on QGIS platform (QGIS Development Team 2009). Figure was prepared with the layers downloaded from the DIVA-GIS website (https://www.diva-gis.org/Data) which is a freely downloadable spatial data source. Hence, it does not require any certification to use its layers. Other layers are created by us which are overlayed on the base map downloaded from the DIVA-GIS website. These layers are processed using the QGIS platform. QGIS Development Team. (2009). QGIS Geographic Information System. Open-Source Geospatial Foundation. http://qgis.org.

Large clusters of wind turbines in Karnataka are located in Chitradurga and Gadag districts. We selected wind farms in these two districts for the current study (Fig. 1). Chitradurga district lies between 14.23° N and 76.39° E. We selected Vani Vilas Sagar (VV Sagar) (24.8 km^2^), Jogimatti hills (100.5 km^2^), and Challkere hills (2.0 km^2^) in the district, having established wind farms. We selected adjoining or the same hills without wind turbines as control sites having a similar habitat, like the wind turbine locations in VV Sagar and Jogimatti (Table 1). Gadag district is located in the north-western part of northern Karnataka, which lies between 15.42° N and 75.62° E. Malaprabha River in the north and Tungabhadra River in the south form the natural boundaries of the district. The district spans over a total geographical area of 4656.0 km^2^. We selected Kappatagudda (320.9 km^2^), Kelur (40.0 km^2^), and Papanasi (27.5 km^2^) wind farm sites in the Gadag district (Table 1) and one site in Kappatagudda without any wind turbines as a control site. Most of the wind turbines were of 0.8–1.25 MW capacity.

**Table 1:** Location and physical characteristics of wind farm and control study sites in Chitradurga and Gadag districts in Karnataka, India.

Sl no.	Parameters	Chitradurga District					Gadag District			
		Wind farm sites			Control sites		Wind farm sites			Control sites
		VV Sagar WS	Jogimatti WS	Challkere WS	VV Sagar CS	Jogimatti CS	Kelur WS	Papanasi WS	Kappatagudda WS	Kappatagudda CS
1	Geocoordinates	13° 49′ 52.45″ N 76°30′ 2.71″ E	14° 11′ 35.09″ N 76° 25′ 5.66″ E	14° 14′ 29.50″ N 76° 26′ 37.65″ E	13° 52′ 0.33″ N 76° 32′ 12.33″ E	14° 10′ 51.39″ N 76° 24′ 13.04″ E	15° 10′ 15.51″ N 75° 45′ 33.67″ E	15° 21′ 25.96″ N 75° 40′ 36.83″ E	15° 14′ 10.30″ N 75° 43′ 14.32″ E	15° 11′ 44.79″ N 75° 45′ 22.19″ E
2	Name and status of the patch	Marikanive RF	Jogimatti RF	Private land	Marikanive RF	Jogimatti RF	Kappatagudda RF	Private land	Kappatagudda RF	Kappatagudda RF
3	Vegetation cover	Dry grassland and scrub forest + mostly *Dodonaea viscosa* cover	Dry grassland + *Acacia* sp. vegetation cover	Dry scrubland	Dry grassland and scrub forest + mostly *Dodonaea viscosa* cover	Dry grassland + *Acacia* sp. vegetation cover	Thorny scrub and dry grassland	Agricultural land	Thorny scrub and dry grassland	Thorny scrub and dry grassland
4	Altitude (m asl)	700–948	700–1010	700–767	700–740	700–1067	660–769	660	660–967	665–900
5	No. of wind turbines	123	18	18	–	–	19	116	203	-
6	Vegetation type	Southern Tropical dry deciduous forest and Southern tropical thorn forest								
6	Average annual temperature	22.1 °C					26.9 °C			
7	Average annual rainfall	573 mm					612.50 mm			
8	Average annual humidity	73.65%					55.97%			
9	Mean wind speed	8.2 km/h					11.4 km/h			

*WS* wind farm site, *CS* control site, *RF* reserved forest.

All the study sites were part of the Reserved Forests, except for Papanasi, which was agricultural land. Largely dry grasslands with scrub forests known as ‘Southern tropical dry deciduous forest and Southern tropical thorn forest’ dominate the landscape^35^. Wind turbines were first deployed at Kappatagudda in 1996, at other sites between 2004 and 2007, and at Papanasi between 2011 and 2014.

### Study design

We monitored the selected wind turbines in VV Sagar, Jogimatti, Challkere, Kappatagudda, Kelur, and Papanasi to evaluate the collision rate of animals with the wind turbines. We assessed the bird diversity at VV Sagar, Jogimatti, and Kappatagudda wind turbine and control sites. Among all the sites, Kappatagudda is one of the large tracts of hill system having one of the oldest (20 years) wind farms. Thus, to understand the long-term impact on animals, we studied the mammalian distribution pattern at Kappatagudda.

### Data collection

#### Methods for carcass search and its persistence

We selected 15 wind turbines at each of VV Sagar WS and Jogimatti WS, 12 wind turbines at Challkere WS in Chitradurga, 15 wind turbines at each of Kappatagudda WS and Papanasi WS, and 14 wind turbines at Kelur WS in Gadag (Table 2). This constituted 7–10% of the total wind turbines on each of the sites, and we randomly selected wind turbines to assess the fatality of animals. In each selected wind turbine for sampling, the searches were made for 30 min/wind turbine within a predefined area of 120 m radius from the wind turbine base and recorded any dead birds/bats once a week from September 2016 to October 2017 in Chitradurga, October 2016 to October 2017 in Gadag. Searches were made by two trained observers walking carefully in a *zig-zag* manner looking for a dead animal on the ground. We recorded the date, time, species, sex, the status of the carcass as fresh or old, distance from wind turbine base, understory cover, and geocoordinates for each detection of a dead animal.

**Table 2:** Search effort and carcasses detected in Chitradurga and Gadag district (VVS = VV Sagar; J = Jogimatti; C = Challkere; K = Kelur; P = Papanasi; KG = Kappatagudda; WS = Wind farm site).

S. no.	Sites	VV S-WS	J-WS	C-WF	KG-WF	P-WF	K-WF
1	No. of wind turbines searched	15	15	12	15	15	14
2	Total no. of days spent	48	48	48	48	48	28
3	Time spent at each turbine (min)	30	30	30	30	30	30
4	Total hours spent^a^	360	360	288	336	360	210
5	No. of visits in a month	4	4	4	4	4	4
6	Area under search at wind turbine (m radius)	120	120	120	120	120	120

^a^Total hours spent = (15 × 48 × 30)/60.

To estimate the intensity of fatality of animals, the mean length of time that the fatalities remain at the location before being removed by other animals, especially by the scavengers is the most important component. Thus, we estimated the average period of the carcass remaining at the site using the methods by following Erickson et al. and Shoenfeld^36,37^. We selected nine wind turbines in VV Sagar WS, ten in Kelur WS, and five in Kappatagudda WS, and kept one dead bird under each wind turbine in the select site, and a camera trap was deployed to focus on the carcasses. The camera trap was programmed to record the date and time on the image for each trigger. We recorded the carcass status and time when the carcass was placed. Every day, visits were made to each site to know the status of the carcass until the carcass disappears from the site.

#### Methods for bird diversity

The bird diversity was assessed between June 2016 and May 2017. We followed two census techniques, viz*.,* fixed-radius point count, and vantage count to count birds at wind turbines and control sites.

Point count: The fixed-radius (40 m) point count method^38^ was followed to estimate the bird abundance and richness at control and wind turbine sites (Table 3). We established 15-point count stations (hereafter ‘points’) in each control and wind turbine site at 200 m intervals. Geocoordinates were recorded using handheld GPS for all the point count stations. Fortnight surveys were conducted at all points except a few sessions, which were interrupted due to rain. We avoided sampling during the heavy rains and unsuitable weather conditions. Thus, the number of temporal replicates in a year/sampling location ranged from 14 to 24 replicates/year. We spent 10 min at each point and counted birds within the fixed radius (40 m) using Nikon Binoculars (8 × 42) between 06:00 and 09:00 h. We also recorded birds by their calls when we were unable to locate the birds. Upon locating birds on a point, we recorded species, a number of individuals, and detection details.

**Table 3:** Sampling effort for bird counting in wind farm and control sites of Karnataka.

No.	Parameters	Wind farm sites			Control sites			Total
		VV Sagar WS	Jogimatti WS	Kappatagudda WS	VV Sagar CS	Jogimatti CS	Kappatagudda CS	
	**Point count**							
1	No. of wind turbines selected	15	15	15	–	–	–	45
2	No. of point count stations	15	15	15	15	15	15	90
3	No. of replications in a month	2	2	2	2	2	2	2
4	Total no. of replications	23	23	14	23	22	24	14–24
5	Time of sampling	06:00–09:00 a.m.						
6	Time spent in each point count station (h)	57.5	57.5	35	57.5	55	60	322.5
	**Raptor sampling**							
8	No. of vantage point count stations	1	1	1	1	1	1	6
9	No. of visits in a month	2	2	2	2	2	2	2
10	Total no of replications	23	23	14	23	22	24	14–24
11	Time of sampling	09:00 a.m.–15:00 p.m. (4.5 h/day)						
12	Time spent in each point count station (h)	103.5	103.5	63	103.5	99	108	580.5

*WS* wind farm site, *CS* control site.

Vantage point count: Raptor count was conducted fortnightly from 09:00 to 15:00 h using the vantage point count method^39^. A vantage point was established for both control and wind turbine sites on a raised place, within the mountain range, to increase the detection of raptors. Elevated points would certainly enhance the visibility of neighboring areas and thus have less chances of missing any raptors. For each raptor’s detection, we recorded the time of observation, species, number of individuals, species behavior (soaring/flying/perched), sighting distance (measured using range finder), flight height, time spent in flight, and distance from the nearest wind turbine.

#### Sampling for mammals

**Figure 2:**
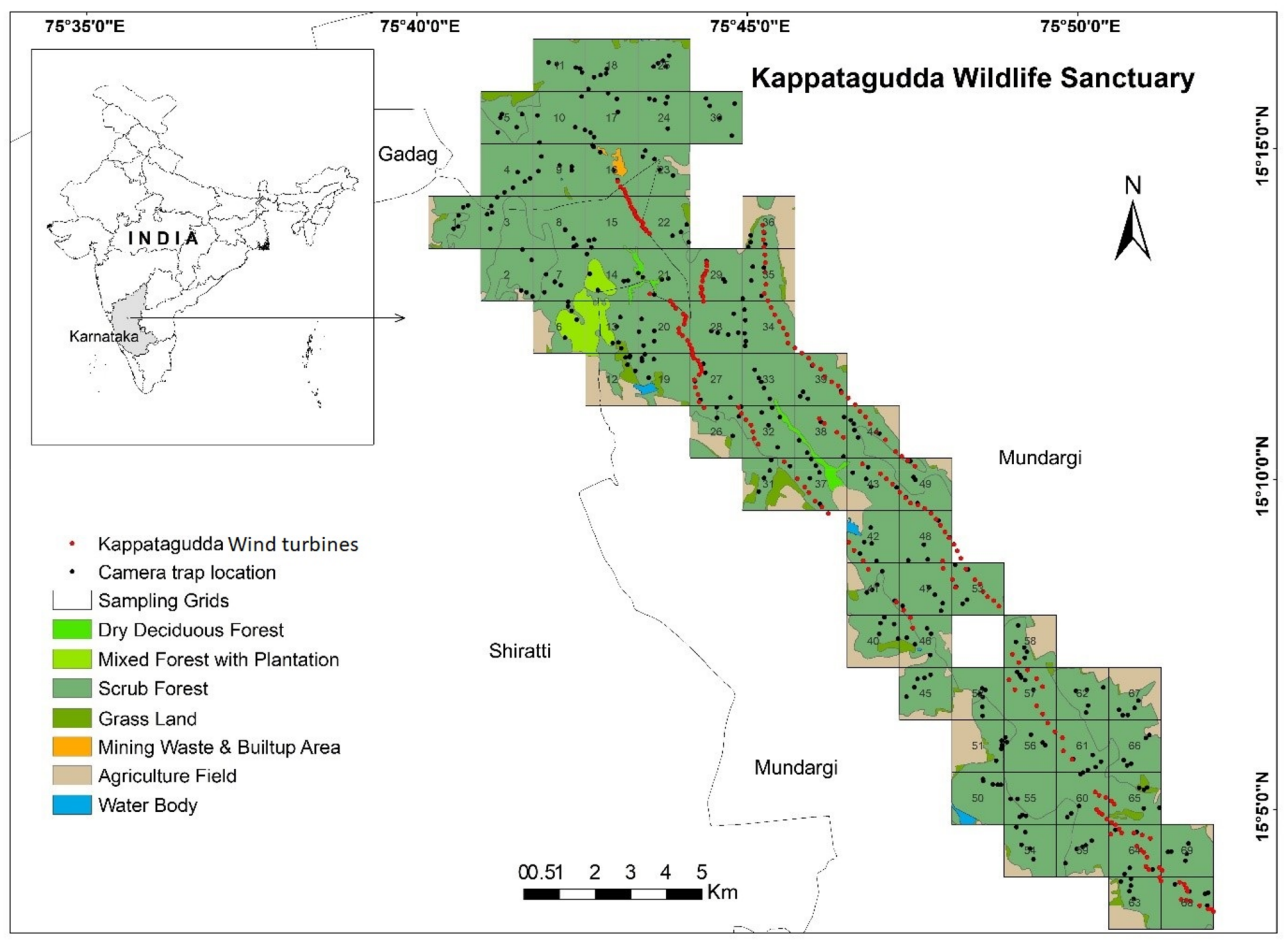
The wind turbines and camera trapping locations in Kappatagudda for mammals in Karnataka. The map is prepared on QGIS platform (QGIS Development Team 2009). Figure was prepared with the layers downloaded from the DIVA-GIS website (https://www.diva-gis.org/Data) which is a freely downloadable spatial data source. Hence, it does not require any certification to use its layers. Other layers are created by us which are overlayed on the base map downloaded from the DIVA-GIS website. These layers are processed using the QGIS platform. QGIS Development Team. (2009). QGIS Geographic Information System. Open-Source Geospatial Foundation. http://qgis.org.

The study was conducted during the dry season (January–May 2018) in the Kappatagudda Wildlife Sanctuary (Area: 320.93 km^2^). We selected an area of ~ 188 km^2^ to study the distribution of mammals in relation to environmental factors and wind turbines (Fig. 2). Since the select hill system consisted of a linear patch (4–5 km width and 17–18 km length) with installed wind turbines, and the major objective was to measure the proportion of habitat occupied by species like Black-naped Hare *Lepus nigricollis* to large animals like Blackbuck *Antilope cervicapra*, we considered a 2 km^2^ area as the smallest unit for sampling. We overlaid 2 km^2^ grids on the polygon of the Kappatagudda Wildlife Sanctuary on a GIS platform using QGIS that provided 94 grid cells. Of the total 94 grid cells, some of the grid cells had a significant proportion of agriculture fields that were excluded from the camera trap sampling due to high human activities. Therefore, we sampled 69 grid cells largely having forest areas in Kappatagudda using the camera trap technique (Fig. 2). We uploaded the grid cells to the global position system (Garmin eTrex60), using this, the sampling grids were realised on the ground. We initially walked the sampling grids to find locations of high mammal activity to deploy the camera traps. We deployed a total of 20 passive infrared motion sensor camera traps (REAP Trail Camera) for the study. We deployed five camera traps at select locations by spacing at least 300 m between the locations in each grid cell for a period of 72 h (three days). We recorded the identity of a camera trap, geocoordinates, and time of deployment for each camera trap deployed. Camera traps were fixed at a height of 100 cm above the ground, and they were set with a trigger gap of 10 s in case animals are continuously present in the camera view. We set the camera trap to record the date and time, and three pictures per trigger. After 3 days of deployment, camera traps were removed, and the images were stored in a separate folder with grid identity and geocoordinates of the location.

Covariates: We considered habitat features and anthropogenic factors as covariates for the species to occupy the space in the study site. We laid a two-kilometer line in each grid cell in a way to cover the entire sampling grid cells. At every 250 m on each line, we employed the point-centered quarter method (PCQ Method)^40^ and collected the data on tree stems more than 10 cm in four directions to the point. We recorded the distance of the tree from the point using a rangefinder (Nikon Forestrypro), girth at breast height (GBH), and height of the tree. We fixed a 10 m radius sampling plot using a rope for every PCQ point. In each sampling plot, we quantified the percent bush cover (< 10 cm GBH of the stems and bushy clumps-BC), the height of those bushes in four corners of the plot (considered the average of that measurement as percent understory height: USH), percent grass (GC), and grass height (the height of the grass was measured using the measuring tape at four quarter of the sampling plot, and the average of that is considered as grass height GH). The forest of the study site was open scrub forests. Measuring the canopy cover using densiometer was not possible. Thus, we walked in four directions from the center of the sampling plot, recorded the canopy connectivity, and visually estimated the canopy cover (CC). We walked back on the same line transect. The dung/droppings of livestock (cattle, goat, and sheep) were counted and recorded on 1.5 m on both sides of the transect and the data was segregated for every 250 m of the line (i.e., eight segments). We recorded evidence of logging or fuelwood extraction on the sampling line. We noted down the number of segments where grazing or tree/branch lopping signs were seen and then multiplied it with 1.25 to convert it into a 10-point scale that represented the intensity of grazing (GR) and logging (TL).

In Kappatagudda, 15 new (0.8 MW) and 15 old (1.25 MW) wind turbines were selected for sampling the noise level. We sampled noise levels at the interval of 250 m away from the wind turbines up to 750 m. The noise level of wind turbines was recorded at ground level using a Digital Sound Level Meter Decibel Logger 30–130 dB. Noise levels highly varied between the old and new wind turbines and gradually decreased as the distance from the wind turbine increased, i.e., 79.40, 63.42, 58.92 dB and 53.47 for the old wind turbines and 62.83, 56.66, 54.95, and 52.59 dB for the new wind turbines at the distance 0, 250, 500 and 750 m from the wind turbines respectively. Thus, we considered the type of wind turbines as the independent covariates (Wind turbine new: WNEW and wind turbine old: WOLD) (Fig. 2). All the wind turbines (old and new) for the Kappatagudda were mapped. They remained between 676 and 977 m asl, with an average of 765 m asl. The grid cells were overlaid on the map of the study site with wind turbine points. The number of each type of wind turbine was counted and considered as covariates for each grid cell (WTOTAL, WNEW, and WOLD).

We traversed and mapped the road networks in the study site using the track mode option in the handheld global position system (Garmin eTrex60). The road network was overlaid on the grid cells, and the total length of the road network was enumerated for each grid cell (total road length-ROAD). The grid cells of the study site were overlaid on the Digital Elevation Model (DEM) of the site and the altitude for 10–15 locations for each grid cell was extracted. We considered the most frequent altitude in the grid cell as a representative (altitude-ALT) for that grid cell.

### Statistical analysis

#### Animal fatality rate

We computed the average period of the carcass remaining at the site using the formula: (T) = Σti/S, where ti is the length of the time carcass remained on the site and S is the total number of carcass placed for the study (Table S1).

We estimated the fatality rate using the formula following Erickson et al.^41^:

$$M = (\underline{N} \cdot \underline{I} \cdot \underline{C})/(\underline{K} \cdot \underline{T} \cdot\underline{ p})$$

where ‘N’ is a total number of wind turbines, ‘I’ is an interval between searches, ‘C’ is a total number of fatalities found during the study, ‘K’ is the number of wind turbines sampled, ‘T’ is the mean length of time fatalities remained in the study area before being removed and ‘p’ denotes searcher efficiency. We considered ‘p’ as 1, which represents the efficiency as maximum since the search area under the turbine was almost barren and clearly visible, and also the observers are trained researchers.

We performed a comprehensive online search of the available literature on Google Scholar (https://scholar.google.com/) using the search words "wind turbine" and a combination of "bird," "bat," "fatality," and "mortality." We compared our findings with the mortality rate due to wind turbines associated with diverse habitat types in coastal and terrestrial habitats.

#### Bird diversity

We treated point count and vantage count data separately for analysis. We pooled all counting data of birds in the area to compute the mean abundance of birds (number of birds counted during each visit against the total number of points) (Table S2). We applied one-way ANOVA to test the difference in species richness and species abundance between wind turbine and control sites. A non-metric multidimensional scaling (nMDS) approach using PAST v.4.03^42^ was applied to elucidate the pattern of species composition in control and wind turbine sites across the sampling locations. This rank-based approach is an indirect gradient analysis that considers dissimilarity or distance matrix to produce ordination. We used the Bray–Curtis dissimilarity index to produce the gradient, and its gradients were assessed through stress values as mentioned in McCune et al.^43^. We ran Analysis of Similarities (ANOSIM) to test the difference in species composition between control and wind turbine sites^44^. Similarly, variation in species composition of birds in different foraging guilds between control and wind turbine sites was assessed using the Multi-Response Permutation Procedure^43^.

#### Occupancy of mammals

Considering the biology of each species and their habitat requirements, we considered parameters and their combinations to be influencing the detection and occupancy of them in each grid cell. Since the study was conducted using camera traps at selected appropriate locations, presuming all animals use the habitat, we considered that none of the selected covariates would influence the capture of the animals that appear in front of the camera traps, i.e., detection probability. Thus, we did not run the model fit for the detection probability, and further models for occupancy were built without keeping any covariates for the detection probability. We hypothesized that the number of wind turbines, tree logging, grazing index, road length would have a negative impact on the occupancy of the species in the grid cells. Considering the habitat requirements, we expected that Four-horned Antelope *Tetracerus quadricornis* may occupy the slopes and hilltops^45^, while altitude may negatively influence the occupancy of Blackbuck^46^, Golden Jackal *Canis aureus*^47^ and Jungle Cat *Felis chaus*^48,49^ conversely, Chinkara *Gazella bennettii*^50,51^ and Black-naped Hare^52^ to occupy the entire study site. However, tree density may negatively influence the occupancy of Blackbuck and Chinkara.

Using the plant data from the Point Cantered Quarter Method (PCQ method), we calculated the tree density (TD), and the basal area (BA) using the formula (GBH)^2^/4π, for each grid cell. We used site-level covariates categorized as ecological variables i.e., bush cover (BC), grass cover (GC), grass height (GH), understory height (USH), canopy cover (CC), tree density (TD), basal area (BA), altitude (ALT), and anthropological variables like grazing index (GR), tree logging index (TL), road length (ROAD), the total number of wind turbines (WTOTAL), number of old wind turbines (WOLD), and number of new wind turbines in the grid cell (WNEW).

To reduce temporal autocorrelation, we considered 12 h as one replication by considering the time required for possible randomization of animal movement. Thus, the total number of replications in 72 h was six. The stored images were carefully checked for the capture of animals. For each capture, we entered the species name, date, time of the capture according to the replication for each camera in a grid. We created a detection matrix for each grid cell using the capture history for each species as ‘1’ = detected and ‘0’ = no detection. We estimated the detection probability (p) and proportion of sites occupied (occupancy: ψ) using the detection histories following maximum likelihood functions^53^ using single-season occupancy modeling in the program PRESENCE 12.9^54^ assuming that the population was closed during sampling. We evaluated the effect of covariates on model parameters on detection probability, and occupancy using logistic models with logit link and binomial error. We built a null model for occupancy and estimated the naïve occupancy for all the species. The naïve estimate was determined by detections of a species in the number of grids divided by the number of grids sampled. We built multiple models using the selected covariates (Table S3). The models were ranked using Akaike’s information criterion (AIC)^55,56^, and the lowest AIC value indicated the best-fit model to the data. If the top model has a model weight ~ 0.9, then considered a parsimonious model^56^. If the model weight of the top model was < 0.9, we considered models with ∆AICc values ≤ 2 of the most parsimonious models, and averaged the model weights of them. We computed model weights and averaging of parameters following Burnham and Anderson^56^. We summed model weights over all the models containing the particular covariate in the select models and ranked them in descending order. We converted the beta coefficients into z-scores by dividing coefficient values with SE to show the effect size.

## Results

### Animal fatality rate

A total of 144 and 124 days were spent on carcass searches in Chitradurga and Gadag districts respectively. We recorded one bird carcass each in Challkere WS, Jogimatti WS, Kelur WS, and Kappatagudda WS, and recorded one bat carcass each in Kappatagudda WS and Challkere WS, and four in Jogimatti WS (Table S4). The carcasses were recorded between 2 and 118 m distances from the wind turbine base. The mean annual birds + bats fatality rate per wind turbine was 0.26 animals per year (Table 4). The Chitradurga (0.33/wind turbine/year) had a higher fatality rate than in Gadag (0.20/wind turbine/year).

**Table 4:** Collision rate of birds and bats in different study sites in Chitradurga and Gadag districts.

District	Study sites	No of wind turbines	Study period (months)	Collision rate/wind turbine/year (birds)	Collision rate/wind turbine/year (bats)	Overall collision rate/wind turbine/year (birds and bats)	Overall collision rate/wind turbine/year (birds and bats) in districts
Chitradurga	VV Sagar WS	15	12	0.00	0.00	0.00	0.33
	Jogimatti WS	15	12	0.13	0.53	0.66	
	Challkere WS	12	12	0.16	0.16	0.33	
Gadag	Kelur WS	14	12	0.21	0.00	0.21	0.20
	Kappatagudda WS	15	7	0.20	0.20	0.40	
	Papanasi WS	15	12	0.00	0.00	0.00	
Overall		86					0.26

*WS* wind farm site.

### Bird diversity

**Figure 3:**
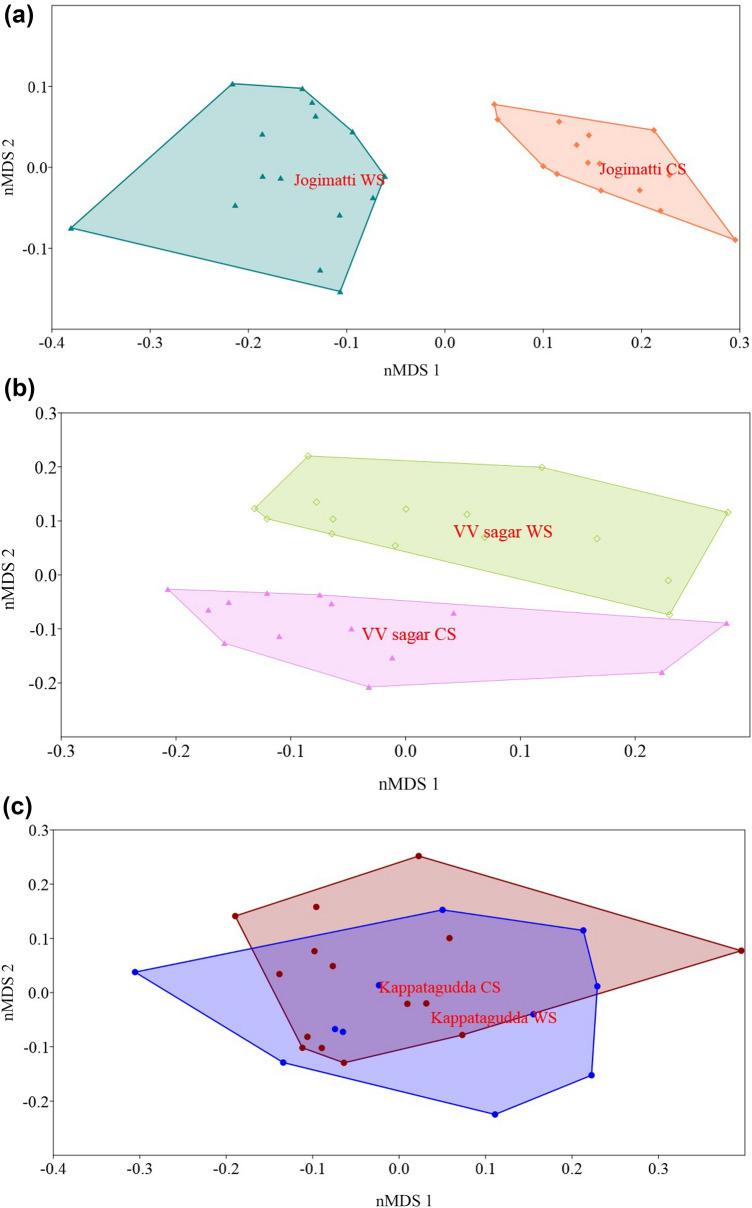
Non-metric multidimensional scaling of species composition between control and wind farm sites: (**a**) Jogimatti, (**b**) VV Sagar, and (**c**) Kappatagudda.

Overall bird richness, mean species richness of birds, and number of unique species (Table 5, Table S2) were relatively higher in control sites over wind turbine sites. The mean abundance of birds among the control (F_2.66_ = 113.38; p < 0.01) and windmill (F_2.57_ = 23.03; p < 0.01) sites were significantly different. However, the mean abundance of birds (Table 5) was two times higher in control sites than in wind turbine sites across the locations (F_1,127_ = 16.14; p < 0.01). The mean abundance and richness of raptors were 1.3 times higher in control sites compared to wind turbine sites (Table 5). The nMDS plot of bird species composition revealed that compared to other sites, Jogimatti and VV Sagar showed a greater difference in species composition between control and wind turbine sites (Fig. 3a, b), while species composition in wind turbine sites at Kappatagudda was a subset of control sites in this location (Fig. 3c). Analysis of Similarity (ANOSIM) indicates that the species composition between control and wind turbine sites (Jogimatti site R = 0.909; p < 0.01; VV Sagar site R = 0.4622; p < 0.01; Kappatagudda site R = 0.114; p < 0.05) were incongruous. The bird species composition of frugivore, granivore, insectivore, omnivore and piscivore guilds were significantly different between control, and wind turbine sites (Table 6).

**Table 5:** Population parameters and functional diversity of birds in different locations of wind farm and control sites in Karnataka State.

Sampling locations	Species richness, mean species richness (SD)	No of unique species and shared species	Mean abundance (SD)	Raptor’s species richness	No. of raptors (h)	Species richness and mean abundance of birds (SD)					
						Frugivore	Granivore	Insectivore	Nectarivore	Omnivore	Piscivore
Jogimatti CS	63 (4.03 ± 2.12)	29, 34	8.06 ± 5.28	6	0.44	6 (3.603 ± 3.088)	8 (0.421 ± 0.93)	38 (3.088 ± 2.984)	3 (0.63 ± 0.972)	4 (0.294 ± 0.792)	1 (0.006 ± 0.078)
Jogimatti WS	39 (1.44 ± 1.22)	5, 34	2.58 ± 2. 78	4	0.30	1 (1.006 ± 1.342)	6 (0.061 ± 0.294)	24 (1.258 ± 2.138)	3 (0.183 ± 0.493)	2 (0.052 ± 0.235)	0 (0 ± 0)
VV Sagar CS	40 (2.28 ± 1.48)	13, 27	3.66 ± 2.88	6	0.48	1 (0.771 ± 1.13)	6 (0.693 ± 1.532)	27 (1.475 ± 1.576)	3 (0.704 ± 1.037)	1 (0.006 ± 0.076)	0 (0 ± 0)
VV Sagar WS	41 (1.46 ± 1.19)	14, 27	2.33 ± 2.37	9	0.44	4 (0.835 ± 1.444)	4 (0.038 ± 0.244)	22 (0.867 ± 1.414)	3 (0.516 ± 0.873)	3 (0.046 ± 0.249)	0 (0 ± 0)
Kappatagudda CS	22 (0.21 ± 0.48)	12, 10	0.42 ± 0.17	10	0.63	1 (0.078 ± 0.466)	4 (0.106 ± 0.629)	16 (0.231 ± 0.854)	1 (0.003 ± 0.053)	0 (0 ± 0)	0 (0 ± 0)
Kappatagudda WS	14 (0.61 ± 0.43)	4, 10	0.31 ± 0.91	6	0.52	1 (0.086 ± 0.51)	2 (0.019 ± 0.168)	8 (0.186 ± 0.757)	1 (0.005 ± 0.069)	0 (0 ± 0)	0 (0 ± 0)
Overall (CS)	81 (0.71 ± 1.60)	34, 47	1.31 ± 3.23	14	0.52	6 (1.433 ± 2.418)	12 (0.402 ± 1.119)	49 (1.557 ± 2.307)	3 (0.437 ± 0.872)	5 (0.096 ± 0.469)	1 (0.002 ± 0.044)
Overall (WS)	58 (0.38 ± 0.89)	11, 47	0.65 ± 1.71	12	0.40	4 (0.726 ± 1.304)	10 (0.042 ± 0.25)	31 (0.858 ± 1.678)	3 (0.269 ± 0.654)	3 (0.038 ± 0.213)	0 (0 ± 0)

*WS* wind farm site, *CS* control site.

**Table 6:** Comparison of functional composition of birds in windmill and control sites.

Feeding guild	Observed delta	Expected delta	*A*	*P*
Frugivore	0.2049	0.2926	0.2998	0.001
Granivore	0.417	0.5327	0.2172	0.001
Insectivore	0.4208	0.4893	0.140	0.001
Nectarivore	0.3019	0.3358	0.1009	0.004
Omnivore	0.3727	0.4362	0.1456	0.001

*delta* average within-group compositional dissimilarity, *A* chance-corrected within-group agreement, *P* proportion of iterations returning dissimilarity values less than those observed.

### Occupancy of mammals

Six species of mammals had more than ten captures, including Blackbuck (12), Chinkara (53), Four-horned Antelope (56), Golden Jackal (130), Jungle Cat (37) and Black-naped Hare (191) (Table 7). The detection probability varied between 0.07 (Blackbuck) and 0.50 (Black-naped Hare), with only the Black-naped Hare and Golden Jackal having a probability > 0.3. The Naïve occupancy of all the species varied between 0.39 and 0.90, except for Blackbuck (0.14). The estimated occupancy for all the species was more than the naïve occupancy except for Golden Jackal. The estimated occupancy in sampled grid cells varied between 0.12 and 0.85, but the mean occupancy (0.50) was less than the naïve occupancy (0.64) for Golden jackal (Tables 8, S3).

**Table 7:** Number of detections, detection probability and naïve occupancy of mammals in Kappatagudda.

Species	Total detection	No. of grids with detection	Detection probability (SE)	Naïve occupancy
Blackbuck	12	10	0.07 ± 0.04_SE_	0.14
Chinkara	53	27	0.27 ± 0.04_SE_	0.41
Four-horned Antelope	56	32	0.20 ± 0.03_SE_	0.51
Golden Jackal	130	44	0.48 ± 0.03_SE_	0.64
Jungle Cat	37	27	0.12 ± 0.03_SE_	0.39
Black-naped Hare	191	62	0.50 ± 0.02_SE_	0.90

**Table 8:** The top models for occupancy of mammals in Kappatagudda.

Species	Model	$\widehat{\psi }$	S*Ê*	AICc	∆AICc	*w*_*i*_	*K*
Blackbuck	*ψ*(WTOTAL), *p*(.)	0.57	0.11	104.22	0.00	0.37	2
	*ψ*(WTOTAL + BC), *p*(.)	0.63	0.17	105.32	1.10	0.21	3
	*ψ*(WTOTAL + ALT), *p*(.)	0.55	0.18	105.81	1.59	0.17	3
Chinkara	*ψ*(WTOTAL + TD), *p*(.)	0.51	0.08	294.45	0.00	0.22	3
	*ψ*(WTOTAL + BC + TD), *p*(.)	0.50	0.11	294.67	0.22	0.19	4
	*ψ*(BC + TD), *p*(.)	0.50	0.08	295.31	0.86	0.14	3
	*ψ*(BC), *p*(.)	0.50	0.06	296.15	1.70	0.09	2
	*ψ*(TD), *p*(.)	0.50	0.05	296.24	1.79	0.09	2
Four-horned Antelope	*ψ*(BC), *p*(.)	0.69	0.10	326.84	0	0.26	2
	*ψ*(.), *p*(.)	0.70	0.11	327.30	0.46	0.21	2
	*ψ*(BC + WNEW), *p*(.)	0.71	0.11	327.59	0.75	0.18	3
	*ψ*(WTOTAL), *p*(.)	0.62	0.09	328.55	1.71	0.11	2
Golden Jackal	*ψ*(ROAD + WOLD), *p*(.)	0.50	0.07	458.60	0.00	0.23	3
	*ψ*(.), *p*(.)	0.65	0.06	458.67	0.07	0.23	2
	*ψ*(ROAD + WTOTAL), *p*(.)	0.50	0.07	459.94	1.34	0.12	3
	*ψ*(ROAD), *p*(.)	0.50	0.05	460.12	1.52	0.11	2
	*ψ*(WOLD + WNEW + ROAD), *p*(.)	0.50	0.09	460.35	1.75	0.10	4
	*ψ*(ROAD + WOLD + GR), *p*(.)	0.50	0.09	460.52	1.92	0.09	4
Jungle Cat	*ψ*(ROAD + WNEW + BC), *p*(.)	0.51	0.11	247.09	0	0.23	4
	*ψ*(ROAD + WTOTAL + BC), *p*(.)	0.50	0.12	247.95	0.86	0.15	4
	*ψ*(BC + WTOTAL), *p*(.)	0.51	0.09	248.61	1.52	0.11	3
	*ψ*(BC + WNEW), *p*(.)	0.51	0.08	248.64	1.55	0.11	3
Black-naped Hare	*ψ*(GR + GC + TC), *p*(.)	0.91	0.06	558.83	0	0.89	5

The total number of wind turbines was in the top models for Blackbuck, Chinkara, Four-horned Antelope, Golden Jackal, and Jungle Cat. The coefficient for wind turbines was negative for all the species except for Four-horned Antelope. Although altitude (*z* = − 0.79) was in the top models, but the total wind turbines remained top with *z* = − 1.53 (Table 9). The top models had no wind turbine covariates for Black-naped Hare, but coefficient estimates for tree cover (*z* = − 1.12) and grass cover (*z* = − 1.29) were negative, while grazing (*z* = 1.50) was positive (Table 9). Percent bush cover was included in the top models of Blackbuck, Chinkara, Four-horned Antelope, and Jungle Cat, but coefficient was positive for all the species except for Jungle Cat (*z* = − 2.13). The coefficient of road length and grazing were positive, while all types of wind turbines were negative for Golden Jackal.

**Table 9:** Covariates influencing the mammal occupancy ranked by summed model weights of covariates with a β coefficient and associated standard error.

Species	Covariate	Summed AIC_c_ weight	*β* coefficient	S*Ê*	z-score
Blackbuck	Total wind turbines (WTOTAL)	0.75	− 2.36	1.54	− 1.53
	Bush cover (BC)	0.21	1.27	1.45	0.88
	Altitude (ALT)	0.17	− 1.03	1.29	− 0.80
Chinkara	Tree density (TD)	0.64	− 0.60	0.35	− 1.71
	Bush cover (BC)	0.43	0.48	0.32	1.50
	Total wind turbines (WTOTAL)	0.41	− 0.57	0.33	− 1.73
Four-horned Antelope	Bush cover (BC)	0.44	0.04	0.02	2.00
	New wind turbines (WNEW)	0.18	0.27	0.41	0.66
	Total wind turbines (WTOTAL)	0.11	0.20	0.20	1.00
Golden Jackal	Total road length (ROAD)	0.65	0.61	0.28	2.18
	Old wind turbines (WOLD)	0.42	− 0.52	0.29	− 1.79
	Total wind turbines (WTOTAL)	0.12	− 0.39	0.27	− 1.44
	New wind turbines (WNEW)	0.10	− 0.14	0.27	− 0.52
	Grazing index (GR)	0.09	0.07	0.27	0.26
Jungle Cat	Bush cover (BC)	0.60	− 0.81	0.38	− 2.13
	Total road length (ROAD)	0.39	0.73	0.45	1.62
	New wind turbines (WNEW)	0.34	− 0.75	0.46	− 1.63
	Total wind turbines (WTOTAL)	0.26	− 0.67	0.41	− 1.63
Black-naped Hare	Tree cover (TC)	0.89	− 0.56	0.5	− 1.12
	Grass cover (GC)	0.89	− 0.83	0.64	− 1.30
	Grazing (GR)	0.89	1.29	0.86	1.50

## Discussion

Animal fatality was recorded at all the wind turbine sites. The calculated animal fatality rate was 0.26 animal/wind turbine/year. The bird richness was more in the control sites than in the wind turbine sites, and their composition was either subset or different in the wind turbine sites over the control sites. Wind turbine numbers in the grid cells remained the top determining variable and the relationship was negative for the occupancy of Blackbuck, Chinkara, Golden Jackal, and Jungle Cat, while the relationship was positive for Four-horned Antelope, and the wind turbine had no effect on the occupancy of Black-naped Hare.

The fatality of animals due to collision with the rotor blade of the wind turbine is determined by the composition and diversity of animals in the area, or if the wind farm is located along the migratory flyway of animals, especially birds^57^. Of the 68 wind farms, where the fatality rate was recorded across the globe (Table S5), Urk, Netherlands had the highest collision rate of birds (51.1/turbine/year) which was carried out during migration^58^. About 41 wind farms had a fatality rate of > 1, nearly half of them were being on agricultural land (Table S5). About 29 wind farms had an animal fatality rate < 1, this includes all the Indian sites monitored for animal fatality rate including the current study sites viz. 0.47 in Harapanahalli, Karnataka^59^, Samakhiali, Kutch region in Gujarat^31^, and 0.38 in Jhangi, Gujarat^30^, except Satara in Maharashtra^33^, where the reported fatality rate was 1.9. Globally, four wind farms had no animal fatalities^60–63^. Most of these studies were focused on the fatality of birds to the wind turbines^64,65^, and the seasonality of such fatality. Prevalence of collision of waterbirds and raptors to the wind turbines are demonstrated in the majority of these studies, few studies of them showed the mortality of Griffon Vulture^19^ and Golden Eagles as 0.1/turbine/year^66^. Large birds with low manoeuvrability (such as swans and geese) are generally at greater risk of collision with structures^67^ and species that habitually fly at dawn and dusk or night are perhaps less likely to detect and avoid turbines^68^.

The studies that addressed the mortality due to wind farms were often local-scale aiming to quantify the collision rates of birds with turbines as well as factors involved in influencing interspecific and local variability^69–71^. Studies from Europe showed that the activity of bats at the turbine rotor height is highest during nights with relatively low wind speeds^72–77^. The actual conservation and population-level consequences of reducing fatalities by changing turbine cut-in speed remain unclear, owing to a dearth of information on bat populations, especially for migratory foliage roosting bats^78^. The meta-analysis of collision rate of birds and bats from the developed countries by Thaxter et al.^79^ revealed that migratory strategy, dispersal distance and habitat associations affected the bird collision rate, while dispersal distance influenced the bat collision rate.

Higher population parameters of birds in control sites over wind farms (Tables 5, 6), which is consistent with earlier studies conducted in India^69,80^, could be due to four reasons: collision, displacement due to disturbance, barrier effects, and habitat loss^81,82^. Here, we recorded low avian mortality, thus displacement and habitat loss could be the reasons, partly, for the variations in our study and elsewhere^83,84^. nMDS analysis added mounting evidence that the species composition of birds in the wind farm was either completely different or a subset of that control sites indicating the disappearance of certain species of birds from wind farms. Certain species are tended to avoid the surroundings of wind farms for foraging, nesting, and roosting during the installation and operation, thus reducing the activity of those species in the long-term in the immediate footprint of turbines^85^.

In Kappatagudda, although the variation in elevation is about 300 m, wind turbines were located on the ridge of the hills (between 676 and 977 m asl), with the average elevation of the wind turbine location being 765 m asl. The average elevation of the control site was less than 650 m. Thus, we expected that many mammal species of the region would occupy the entire hill system, with the exception of the Blackbuck, which may avoid higher slopes. Although Blackbucks are known to occur in the plains, they also occupy a wide range of habitats including semi-arid grasslands, open scrub, dry river beds, grassy forest clearings, and open forests^86–91^. Although altitude was one of the predictive variables, the total wind turbines remained at the top and negatively affected the occupancy of Blackbuck in Kappatagudda. Similarly, Chinkara is also known to occur in the plains but prefers the open scrublands and thinly wooded forests^92–94^ and elevations of up to 1200 m asl^90^. Chinkara is confined to thinly forested or scrub forests in Karnataka including the hilly terrain (e.g., Yadahalli Wildlife Sanctuary)^94,95^. However, the increased bush cover determined the occupancy of Chinkara, but the tree density and total wind turbines negatively affected the occupancy in Kappatagudda. Golden Jackal and Jungle Cat are highly adaptable to live in various ecological conditions, thus making them globally widespread^96,97^. In Kappatagudda, the road network increased the occupancy of Golden Jackal and Jungle Cat, while wind turbines negatively determined their occupancy.

Four-horned Antelope is the forest antelope and occurs in the tree savanna deciduous forests^98^ and undulating hilly terrain^91^. Similarly, the occupancy of the Four-horned Antelope was confined to the slopes and ridges of the hill system, with the increase in the bush cover and wind turbines determining their occupancy in Kappatagudda. Since the number of wind turbines was high on the ridges of the slopes, the presence of wind turbine probably has emerged as the predictor. The Black-naped Hare is a habitat generalist and known to occur anywhere from forests to cropland^91^. Anoop et al.^52^ reported that the abundance of Black-naped Hare is more in the wind turbine area over the control site due to less predatory pressure. However, the grazed habitat determined the occupancy of Black-naped Hare, while the increased tree cover and grass cover were negative. This indicates that wind turbines had no impact on the occupancy of the Black-naped Hare in Kappatagudda.

The number of wind turbines in the grid cell (either total or old or new) remained one of the predictive variables that the relationship was negative for Blackbuck, Chinkara, Golden Jackal and Jungle Cat, where it was positive for Four-horned Antelope, but did not predict the occupancy of Black-naped Hare. Although the abundance of wind turbines did not play a major role in the occupancy of a few species, the avoidance of wind turbines by many mammals is apparent, which is of major concern to the management of the forested area where the wind turbines are established.

The study was conducted in the established wind farms where we had no data on earlier biodiversity, thus the conclusion drawn by comparing the bird diversity in wind farm sites with the control site may be considered as one of the caveats in the current study. However, the selected control sites are part of the same respective hill systems having the same habitat conditions with slight differences. Thus, in this context, we consider comparing the bird diversity is the only way that we can have a better understanding of the responses of birds to the wind turbines. In a nutshell, the direct collision of animals to the turbine blades is negligible in these long-established wind farms, however, the disappearance of birds and mammals in the wind farms is evident. Thus, just considering the collision rate or fatality rate may not be sufficient and may not be a true indicator while assessing the impact of wind turbines on animals in the natural habitat too in the long-established wind farms. We suggest retaining a portion of hill regions or natural habitat untouched in the wind turbine-dominated terrain as refugia for animals. The long-term monitoring of the biodiversity around the existing wind farms is lacking in many landscapes. The studies on raptors and their food resources like rodents, reptiles are essential to understanding the consequences of turbines. The findings from these studies would help to manage and mitigate the impact caused by wind turbines. The establishment of wind farms may rise in the future, not only in India, even globally. Prior to the turbine installation or before licensing for the new wind farms especially in the forested areas and also in the vicinity of the potential forest patches or next to wetlands^79^, it is suggested to have a critical evaluation of animal diversity especially the birds and their seasonal movements, occupancy and abundance of mammals, and possible impact on them.

## Supplementary Information

Supplementary Information.

## Data Availability

Data is provided in supplementary tables. Any additional information or data is expected that shall be provided based on the demand.
