# Supplementary material for: Responses of birds and mammals to long-established wind farms in India
Source: Sci Rep. 2022 Jan 25;12:1339. doi: 10.1038/s41598-022-05159-1 (PMC8789773; doi:10.1038/s41598-022-05159-1)
Supplement: Supplementary file 1 — Supplementary Information. [file 41598_2022_5159_MOESM1_ESM.pdf]

Table S1. Estimation of the duration of time that each carcass remained on the site (The duration of time each carcass remains on the site before being removed by Scavenger (T) was: Formula (T)= Σti/S, (Ti - The length of a time carcass remained on the site; S - The total number of carcass kept for the study).

Parameters	Chitradurga	Gadag
ti	27 days	18 days
S	Nine samples plotted	Nine samples plotted
T	27/9 = **3 days**	18/9 = **2 days**

The estimated average time for the carcass being removed from the site was three days in the Chitradurga district and two days in Gadag district. Three species mainly Grey Mongoose *Herpestes edwardsii*, Ruddy Mongoose *Herpestes smithii*, and Common Palm Civet *Paradoxurus hermaphroditus* scavenged on the carcasses in Chitradurga, and stray dogs, Golden Jackal *Canis aureus*, Ruddy Mongoose, Jungle Cat *Felis chaus* and Wild Pig *Sus scrofa* scavenged on the carcass in Gadag district.

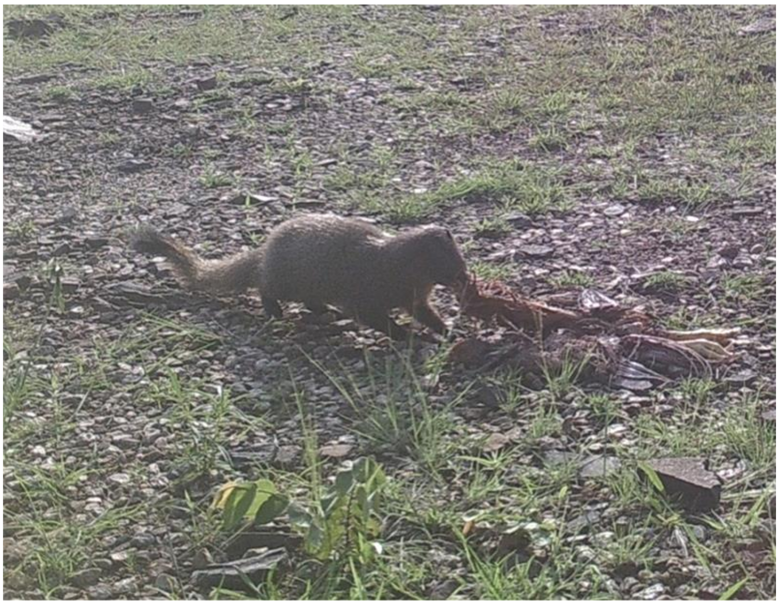

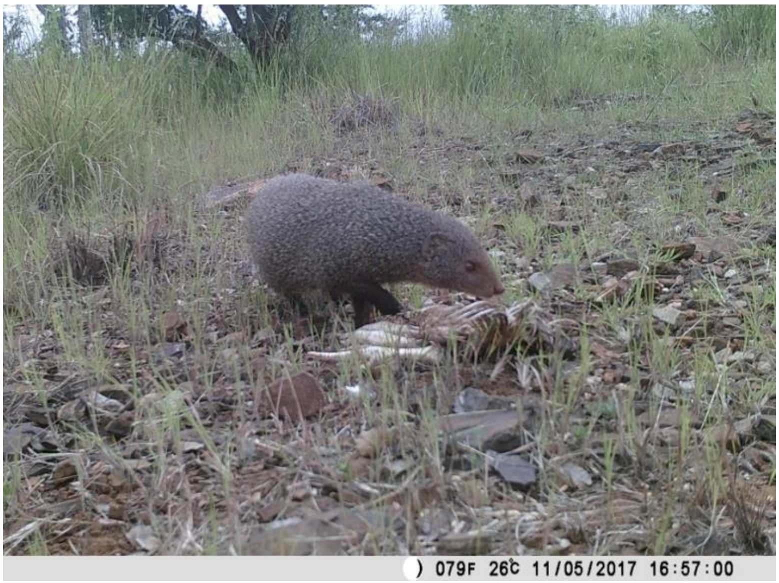

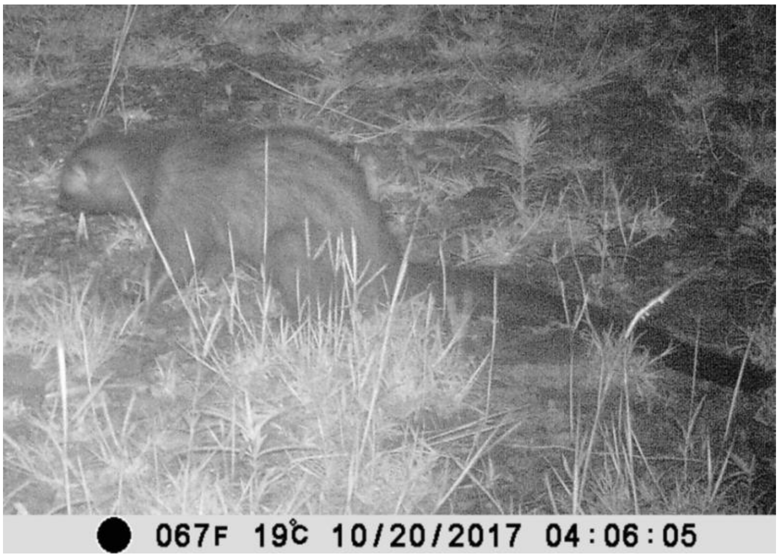

Carcass removal by Ruddy Mongoose, Common Mongoose, and Common Palm Civet

Table S2. List of bird species recorded in wind farm and control sites of Chitradurga and Gadag districts

Order	English Name[a]	Scientific Name	Migratory Status [b]	Abundance	IUCN category[c]	Guild[d]	Wind farm sites[e]			Control sites		
							VVS	JG	KP	VVS	JG	KP
Galliformes	Indian Peafowl	*Pavo cristatus*	R	Common	LC	O	-	-	-	-	+	-
Galliformes	Grey Junglefowl	*Gallus sonneratii*	R	Uncommon	LC	O	-	-	-	-	+	-
Galliformes	Grey Francolin	*Francolinus pondicerianus*	R	Common	LC	G	-	+	-	-	+	-
Galliformes	Jungle Bush Quail	*Perdicula asiatica*	R	Uncommon	LC	G	-	+	-	-	+	-
Ciconiiformes	Asian Openbill	*Anastomus oscitans*	R/LM	Common	LC	Invt	+	-	-	-	-	-
Pelecaniformes	Intermediate Egret	*Ardea intermedia*	R/LM	Common	LC	P	-	-	-	-	+	-
Falconiformes	Common Kestrel	*Falco tinnunculus*	WM	Common	LC	C	+	+	+	-	-	+
Falconiformes	Red-necked Falcon	*Falco chicquera*	R	Common	NT	C	+	-	-	-	-	+
Falconiformes	Peregrine Falcon	*Falco peregrinus*	WM	Common	LC	C	-	-	-	-	-	+
Accipitriformes	Black Kite	*Milvus migrans*	R	Common	LC	C	-	-	+	-	-	+
Accipitriformes	Booted Eagle	*Hieraaetus pennatus*	WM	Common	LC	C	-	-		+	-	-
Accipitriformes	Brahminy Kite	*Haliastur indus*	R	Common	LC	C	-	-	+	-	-	-
Accipitriformes	Eurasian Sparrowhawk	*Accipiter nisus*	WM	Uncommon	LC	C	-	-	-	-	+	-
Accipitriformes	Oriental Honey Buzzard	*Pernis ptilorhynchus*	R	Common	LC	C	+	-	-	+	+	+
Accipitriformes	Short-toed Snake Eagle	*Circaetus gallicus*	R	Common	LC	C	+	-	+	+	+	+
Accipitriformes	Egyptian Vulture	*Neophron percnopterus*	R	Common	EN	Sca	+	-	-	+	-	+
Accipitriformes	Black-winged Kite	*Elanus caeruleus*	R	Common	LC	C	-	+	-	-	+	-
Accipitriformes	Black Eagle	*Ictinaetus malaiensis*	R	Common	LC	C	+	+	+	-	+	+
Accipitriformes	Steppe Eagle	*Aquila nipalensis*	WM	Rare	EN	C	+	-	-	-	-	-
Accipitriformes	Shikra	*Accipiter badius*	R	Common	LC	C	+	+	+	+	+	+
Accipitriformes	White-eyed Buzzard	*Butastur teesa*	R/LM	Uncommon	LC	C	+	-	-	+	-	-
Accipitriformes	Bonelli's Eagle	*Aquila fasciata*	R/LM	Common	LC	C	-	-	-	+	-	+

Caprimulgiformes	Indian Nightjar	*Caprimulgus asiaticus*	R	Uncommon	LC	I	-	-	-	+	-	-
Caprimulgiformes	Asian Palm Swift	*Cypsiurus balasiensis*	R	Common	LC	I	-	+	+	+	+	+
Caprimulgiformes	Indian House Swift	*Apus affinis*	R	Common	LC	I	+	+	-	+	+	-
Charadriiformes	Red-wattled Lapwing	*Vanellus indicus*	R	Common	LC	I	-	-	-	+	-	-
Columbiformes	Laughing Dove	*Streptopelia senegalensis*	R	Common	LC	G	+	+	-	+	+	+
Columbiformes	Spotted Dove	*Streptopelia chinensis*	R	Common	LC	G	-	+	-	+	+	-
Columbiformes	Eurasian Collared Dove	*Streptopelia decaocto*	R	Common	LC	G	+	-	-	+	+	-
Psittaciformes	Rose-ringed Parakeet	*Psittacula krameri*	R	Common	LC	F	+	-	-	+	+	-
Psittaciformes	Plum-headed Parakeet	*Psittacula cyanocephala*	R	Uncommon	LC	F	-	-	-	+	-	-
Cuculiformes	Asian Koel	*Eudynamys scolopaceus*	R	Common	LC	F	+	-	-	-	+	-
Cuculiformes	Greater Coucal	*Centropus sinensis*	R	Common	LC	O	+	+	-	-	+	-
Cuculiformes	Blue-faced Malkoha	*Phaenicophaeus viridirostris*	R	Uncommon	LC	I	-	+	-	-	+	+
Cuculiformes	Sirkeer Malkoha	*Taccocua leschenaultii*	R	Common	LC	I	+	+	-	+	+	+
Coraciiformes	Indian Roller	*Coracias benghalensis*	R	Common	LC	I	+	+	-	+	+	-
Coraciiformes	Green Bee-eater	*Merops orientalis*	R	Common	LC	I	+	+	+	+	+	+
Bucerotiformes	Common Hoopoe	*Upupa epops*	R	Common	LC	I	-	-	-	-	+	-
Piciformes	Coppersmith Barbet	*Psilopogon haemacephalus*	R	Common	LC	F	-	-	-	-	+	-
Passeriformes	Indian Pitta	*Pitta brachyura*	R/WM	Uncommon	LC	I	-	+	-	+	+	-
Passeriformes	Common Woodshrike	*Tephrodornis pondicerianus*	R	Common	LC	I	+	+	-	+	+	-
Passeriformes	Common Iora	*Aegithina tiphia*	R	Common	LC	I	+	+	-	+	+	-

Passeriformes	Small Minivet	*Pericrocotus cinnamomeus*	R	Common	LC	I	+	-	-	+	+	-
Passeriformes	Black Drongo	*Dicrurus macrocercus*	R	Common	LC	I	+	+	+	+	+	+
Passeriformes	Ashy Drongo	*Dicrurus leucophaeus*	R/WM	Common	LC	I	+	+	-	+	+	-
Passeriformes	White-bellied Drongo	*Dicrurus caerulescens*	R	Uncommon	LC	I	+	-	-	+	+	-
Passeriformes	White-browed Fantail	*Rhipidura aureola*	R	Common	LC	I	-	-	-	-	+	-
Passeriformes	Black-naped Monarch	*Hypothymis azurea*	R	Uncommon	LC	I	-	-	-	-	+	-
Passeriformes	Indian Paradise-flycatcher	*Terpsiphone paradisi*	R	Uncommon	LC	I	-	-	-	-	+	-
Passeriformes	Rufous Treepie	*Dendrocitta vagabunda*	R	Common	LC	O	+	+	-	-	+	-
Passeriformes	Cinereous Tit	*Parus cinereus*	R	Uncommon	LC	I	+	-	-	-	+	-
Passeriformes	Dusky Crag Martin	*Ptyonoprogne concolor*	R	Common	LC	I	+	-	-	-	-	-
Passeriformes	Red-rumped Swallow	*Cecropis daurica*	R	Common	LC	I	+	+	+	+	+	+
Passeriformes	Barn Swallow	*Hirundo rustica*	R/WM	Common	LC	I	+	+	-	+	+	-
Passeriformes	Rufous-tailed Lark	*Ammomanes phoenicura*	R	Common	LC	I	-	+	+	+	-	+
Passeriformes	Indian Bushlark	*Mirafra erythroptera*	R	Common	LC	I	-	-	+	-	-	-
Passeriformes	Sykes's Lark	*Galerida deva*	R	Uncommon	LC	I	-	-	-	+	-	-
Passeriformes	Ashy-crowned Sparrow Lark	*Eremopterix griseus*	R	Common	LC	G	-	-	-	-	-	+
Passeriformes	Red-vented Bulbul	*Pycnonotus cafer*	R	Common	LC	F	+	+	+	+	+	+
Passeriformes	White-browed Bulbul	*Pycnonotus luteolus*	R	Common	LC	F	-	-	-	-	+	-
Passeriformes	Red-whiskered Bulbul	*Pycnonotus jocosus*	R	Common	LC	F	-	-	-	-	+	-

Passeriformes	Yellow-throated Bulbul	*Pycnonotus xantholaemus*	R	Uncommon	VU	F	+	-	-	-	-	-
Passeriformes	Grey-breasted Prinia	*Prinia hodgsonii*	R	Common	LC	I	+	+	-	+	+	-
Passeriformes	Ashy Prinia	*Prinia socialis*	R	Common	LC	I	+	+	-	+	+	-
Passeriformes	Plain Prinia	*Prinia inornata*	R	Common	LC	I	+	+	-	+	+	-
Passeriformes	Jungle Prinia	*Prinia sylvatica*	R	Common	LC	I	+	+	-	+	+	-
Passeriformes	Common Tailorbird	*Orthotomus sutorius*	R	Common	LC	I	+	+	-	+	+	+
Passeriformes	Blyth's Reed Warbler	*Acrocephalus dumetorum*	WM	Common	LC	I	-	-	-	-	+	-
Passeriformes	Clamorous Reed Warbler	*Acrocephalus stentoreus*	WM	Uncommon	LC	I	-	-	-	-	-	+
Passeriformes	Greenish Warbler	*Phylloscopus trochiloides*	R/WM	Common	LC	I	+	+	-	-	+	-
Passeriformes	Tawny-bellied Babbler	*Dumetia hyperythra*	R	Common	LC	I	-	-	-	-	+	-
Passeriformes	Yellow-billed Babbler	*Argya affinis*	R	Common	LC	I	+	+	-	+	+	-
Passeriformes	Large Grey Babbler	*Argya malcolmi*	R	Common	LC	I	-	+	-	-	+	-
Passeriformes	Common Babbler	*Argya caudata*	R	Common	LC	I	-	+	-	-	-	-
Passeriformes	Yellow-eyed Babbler	*Chrysomma sinense*	R	Ucommon	LC	I	-	-	-	-	-	+
Passeriformes	Lesser Whitethroat	*Curruca curruca*	WM	Uncommon	LC	I	-	-	-	-	+	-
Passeriformes	Common Myna	*Acridotheres tristis*	R	Common	LC	O	+	-	-	+	-	-
Passeriformes	Orange-headed Thrush	*Geokichla citrina*	R	Common	LC	I	-	-	-	-	+	-
Passeriformes	Oriental Magpie Robin	*Copsychus saularis*	R	Common	LC	I	-	-	-	-	+	-
Passeriformes	Indian Robin	*Copsychus fulicatus*	R	Common	LC	I	+	+	-	+	+	+
Passeriformes	Pied Bushchat	*Saxicola caprata*	R	Common	LC	I	-	-	-	-	+	-
Passeriformes	Blue Rock Thrush	*Monticola solitarius*	WM	Common	LC	I	-	-	+	-	-	+
Passeriformes	Asian Brown Flycatcher	*Muscicapa dauurica*	R/WM	Uncommon	LC	I	-	-	-	-	+	-

Passeriformes	Tickell's Blue Flycatcher	*Cyornis tickelliae*	R	Common	LC	I	-	-	-	-	+	-
Passeriformes	Jerdon's Leafbird	*Chloropsis jerdoni*	R	Common	LC	F	+	-	-	-	+	-
Passeriformes	Pale-billed Flowerpecker	*Dicaeum erythrorhynchos*	R	Common	LC	N	+	+	-	+	+	+
Passeriformes	Thick-billed Flowerpecker	*Dicaeum agile*	R	Common	LC	N	-	-	-	+	+	-
Passeriformes	Purple-rumped Sunbird	*Leptocoma zeylonica*	R	Common	LC	N	+	+	-	+	+	-
Passeriformes	Purple Sunbird	*Cinnyris asiaticus*	R	Common	LC	N	+	+	+	+	+	-
Passeriformes	Yellow-throated Sparrow	*Gymnoris xanthocollis*	R	Common	LC	G	-	-	+	-	-	+
Passeriformes	Indian Silverbill	*Euodice malabarica*	R	Common	LC	G	-	+	+	+	-	+
Passeriformes	Scaly-breasted Munia	*Lonchura punctulata*	R	Common	LC	G	-	-	-	-	+	-
Passeriformes	White-rumped Munia	*Lonchura striata*	R	Common	LC	G	-	+	-	-	-	-
Passeriformes	Red Munia	*Amandava amandava*	R	Common	LC	G	-	-	-	-	+	-
Passeriformes	Paddyfield Pipit	*Anthus rufulus*	R	Common	LC	I	-	-	-	-	-	+
Passeriformes	Blyth's Pipit	*Anthus godlewskii*	WM	Uncommon	LC	I	-	-	-	-	-	+
Passeriformes	Richard's Pipit	*Anthus richardi*	WM	Uncommon	LC	I	-	-	+	-	-	+
Passeriformes	Long-billed Pipit	*Anthus similis*	R/WM	Uncommon	LC	I	-	-	-	+	-	+
Passeriformes	Red-headed Bunting	*Emberiza bruniceps*	WM	Uncommon	LC	G	+	-	-	-	-	-

[a]= Praveen et al. (2021) nomenclature was followed here (Checklist of birds of India v5.0);

[b]=R=Resident; LM=Local migrant; WM=Winter Migrant - migratory status of birds for India were compiled from Birdlife International;

[c]=LC=Least Concern; NT=Near-threatened; VU= Vulnerable; EN=Endangered (Source: Birdlife International);

[d]= G=Granivore; F=Frugivore; I=Insectivore; C=Carnivore; O=Omnivore; Inv=Invertebrate; N=Nectarivore; S=Scavenger (Source: Wilman et al 2014 & we used our observations for some species)

[e]= VVS=VV Sagar; JG=Jogimatti; KP= Kappatagudda

Praveen J., Jayapal, R., & Pittie, A., 2021. Checklist of the birds of India (v5.0). Website: http://www.indianbirds.in/india/ [Date of publication: 29 March, 2021].

Wilman, H., Belmaker, J., Simpson, J., de la Rosa, C., Rivadeneira, M. M., & Jetz, W. (2014). EltonTraits 1.0: Species-level foraging attributes of the world's birds and mammals: Ecological Archives E095-178. *Ecology*, *95*(7), 2027-2027.

Table S3. All the models for occupancy of mammals in Kappatagudda

Species	Model	$\hat{\psi}$	$S\hat{E}$	AICc	ΔAICc	w_i	K
Blackbuck	ψ(WTOTAL), p(.)	0.57	0.11	104.22	0	0.37	2
	ψ(WTOTAL+BC), p(.)	0.63	0.17	105.32	1.1	0.21	3
	ψ(WTOTAL+ALT), p(.)	0.55	0.18	105.81	1.59	0.17	3
	ψ(WTOTAL+BC+ALT), p(.)	0.5	0.22	106.83	2.61	0.1	4
	ψ(BC), p(.)	0.49	0.11	108.39	4.17	0.05	2
	ψ(ALT), p(.)	0.51	0.13	109.99	5.77	0.02	2
	ψ(GC), p(.)	0.5	0.11	110.39	6.17	0.02	2
	ψ(GR), p(.)	0.5	0.15	110.59	6.37	0.02	2
	ψ(USH), p(.)	0.51	0.04	110.94	6.72	0.01	2
	ψ(TL), p(.)	0.5	0.13	110.98	6.76	0.01	2
	ψ(.), p(.)	0.39	0.23	111.06	6.84	0.01	2
	ψ(TD), p(.)	0.5	0.11	111.08	6.86	0.01	2
Chinkara	ψ(WTOTAL+TD), p(.)	0.51	0.08	294.45	0	0.22	3
	ψ(WTOTAL+BC+TD), p(.)	0.5	0.11	294.67	0.22	0.19	4
	ψ(BC+TD), p(.)	0.5	0.08	295.31	0.86	0.14	3
	ψ(BC), p(.)	0.5	0.06	296.15	1.7	0.09	2
	ψ(TD), p(.)	0.5	0.05	296.24	1.79	0.09	2
	ψ(WTOTAL), p(.)	0.5	0.06	296.59	2.14	0.07	2
	ψ(TL), p(.)	0.5	0.06	297.33	2.88	0.05	2
	ψ(USH), p(.)	0.46	0.07	297.41	2.96	0.05	2
	ψ(TH), p(.)	0.5	0.06	298.24	3.79	0.03	2
	ψ(ALT), p(.)	0.5	0.06	298.98	4.53	0.02	2
	ψ(GR), p(.)	0.5	0.06	298.99	4.54	0.02	2
	ψ (.), p(.)	0.48	0.03	299.17	4.72	0.02	2
Four-horned Antelope	ψ(BC), p(.)	0.69	0.1	326.84	0	0.26	2
	ψ(.), p(.)	0.7	0.11	327.3	0.46	0.21	2
	ψ(BC+WNEW), p(.)	0.71	0.11	327.59	0.75	0.18	3
	ψ(WTOTAL), p(.)	0.62	0.09	328.55	1.71	0.11	2
	ψ(WNEW), p(.)	0.55	0.03	329.7	2.86	0.06	2
	ψ(WOLD+WNEW), p(.)	0.62	0.08	330.16	3.32	0.05	3
	ψ(GR), p(.)	0.59	0.08	330.26	3.42	0.05	2
	ψ(GR+WNEW), p(.)	0.63	0.1	330.47	3.63	0.04	3
	ψ(WOLD), p(.)	0.55	0.05	330.76	3.92	0.04	2
Golden Jackal	ψ(ROAD+WOLD), p(.)	0.5	0.07	458.6	0	0.23	3
	ψ(.), p(.)	0.65	0.06	458.67	0.07	0.23	2
	ψ(ROAD+WTOTAL), p(.)	0.5	0.07	459.94	1.34	0.12	3
	ψ(ROAD), p(.)	0.5	0.05	460.12	1.52	0.11	2
	ψ(WOLD+WNEW+ROAD), p(.)	0.5	0.09	460.35	1.75	0.1	4
	ψ(ROAD+WOLD+GR), p(.)	0.5	0.09	460.52	1.92	0.09	4
	ψ(WOLD), p(.)	0.5	0.05	462	3.4	0.04	2
	ψ(WTOTAL), p(.)	0.5	0.05	463.65	5.05	0.02	2

	ψ(BC), *p*(.)	0.5	0.05	463.82	5.22	0.02	2
	ψ(TD), *p*(.)	0.5	0.05	464.54	5.94	0.01	2
	ψ(ALT), *p*(.)	0.5	0.05	464.62	6.02	0.01	2
	ψ(GR), *p*(.)	0.5	0.05	464.64	6.04	0.01	2
	ψ(WNEW), *p*(.)	0.5	0.04	464.67	6.07	0.01	2
Jungle Cat	*ψ*(ROAD+WNEW+BC), *p*(.)	0.51	0.11	247.09	0	0.23	4
	ψ(ROAD+WTOTAL+BC), *p*(.)	0.5	0.12	247.95	0.86	0.15	4
	ψ(BC+WTOTAL), *p*(.)	0.51	0.09	248.61	1.52	0.11	3
	ψ(BC+WNEW), *p*(.)	0.51	0.08	248.64	1.55	0.11	3
	ψ(BC), p(.)	0.5	0.06	249.34	2.25	0.08	2
	ψ(ROAD+BC), *p*(.)	0.5	0.1	249.38	2.29	0.07	3
	ψ(WNEW+ROAD), *p*(.)	0.5	0.09	249.45	2.36	0.07	3
	ψ(ROAD), *p*(.)	0.5	0.07	251.13	4.04	0.03	2
	ψ(WOLD+BC), *p*(.)	0.5	0.1	251.2	4.11	0.03	3
	ψ(WNEW), *p*(.)	0.51	0.05	251.37	4.28	0.03	2
	ψ(.), *p*(.)	0.7	0.17	251.46	4.37	0.03	2
	ψ(WTOTAL), *p*(.)	0.5	0.07	252.35	5.26	0.02	2
	ψ(ALT), *p*(.)	0.5	0.07	253.09	6	0.01	2
	ψ(GR), *p*(.)	0.5	0.07	253.28	6.19	0.01	2
	ψ(WOLD), *p*(.)	0.5	0.07	253.54	6.45	0.01	2
	ψ(TD), *p*(.)	0.5	0.07	253.55	6.46	0.01	2

Table S4. The details of the carcasses found around the windmill

Date	Site	Latitude	Longitude	Species	No.	DFT
02/09/2016	Challkere	14.240700	76.444188	Black Kite	1	20.5
23/11/2016	Jogimatti	14.199187	76.422598	Spotted Owlet	1	118.7
26/10/2017	Jogimatti	14.19997	76.42487	Bat	1	15.0
26/10/2017	Jogimatti	14.19328	76.41809	Bat	1	10.0
26/10/2017	Jogimatti	14.19684	76.41661	Bat	1	4.0
26/10/2017	Jogimatti	14.20242	76.41563	Bat	1	11.0
27/10/2017	Challkere	14.23864	76.44584	Bat	1	2.0
13/05/2017	Kelur	15.177149	75.751623	Black Drongo	1	22.9
05/07/2017	Kappadagudda	15.233501	75.721822	Cinnamon Bittern	1	27.0
07/10/2017	Kappadagudda	15.240455	75.718044	Bat	1	6.5

DFT= Distance from the windmill base (m). LC-Least Concern, IUCN-International Union for the Conservation of Nature.

Table S5. Fatality rate of birds and bats in different regions

No.	Site	Habitat	Collision Rate/ Turbine /Year	Source
1	Urk, Netherlands	Coastal-on dyke wall	51.1	(Winkelman 1989)
2	West Virginia, USA	Forest area	42.7	(Kerns & Kerlinger 2004)
3	Isthmus of Tehuantepec, Mexico	Coastal plains	7.70-37.23	(Cabrera-Cruz et al. 2020)
4	Northern Spain	Lowlands, mainly agricultural lands.	29.67	(Camina 2012)
5	Jura Mountains, Northwest, Switzerland	Forest, grassland and pastures	29.22	(Aschwanden et al. 2018)
6	Netherlands	Flat, large scale open agricultural areas	28	(Krijgsveld et al. 2009)
7	Horicon Marsh National Wildlife Refuge, USA	Agricultural land	26.25	(Grodsky et al. 2012)
8	Zeebrugge, Belgium	Coastal shoreline	4-23	(Everaert et al. 2002)
9	Eastern Tennessee, USA	Plantation & mixed-mesophytic forests	21.4	(Fiedler 2004)
10	Flanders, Belgium	Agricultural land, breeding colony of terns and gulls	21	(Everaert 2014)
11	Southwestern Alberta, Canada	Agriculture and native mixed-grasslands	20.56	(Baerwald & Barclay 2011)
12	Zeebrugge, Belgium	Sea coast	19.1-20.9	(Everaert & Stienen 2007)
13	Summer View Windfarm Alberta, Canada	Land relatively broad, level plateau within the Grassland Natural Region.	1.9-18.48	(Brown & Brenda 2006a)
14	Coega, Port Elizabeth, Eastern Cape, South Africa	Dense evergreen bush at river side	18	(Doty & Martin 2013)
15	Canada	Agricultural land	15.5 ± 3.8	(Zimmerling & Francis 2016)
16	Oosterbierum, Netherlands	Coastal-on dyke wall	14.6	(Winkelman 1992)
17	North-east Netherlands and northwestern Germany	Agricultural land	9.7	(Schaub et al. 2020)
18	Wisconsin, USA	Agricultural land	9.49	(Grodsky et al. 2013)
19	Canada	Various	8.2 ± 1.4	(Zimmerling et al. 2013)
20	Southeastern United States	Coal surface mine	7.27	(Nicholson et al. 2005)
21	Worth County, Iowa	Grassland and deciduous woodlots associated with farmsteads	4.45-7.14	(Jain et al. 2011)

22	Northeast Iowa	Mixed agriculture-prairie-forest landscape	7	(Crotty et al. 2014)
23	Basque Country, Spain	Inland hills	5-7	(Onrubia et al. 2002)
24	Minnesota, USA	Forest hills	1.47-6.67	(Johnson et al. 2002)
25	Wellington south coast, New Zealand	Hilly coast	4.64-5.83	(Bull et al. 2013)
26	Northeastern United States	--	5.58	(Choi et al. 2020)
27	Dakotas, USA	Agricultural land and grassland	4.38-5.43	(Graff et al. 2014)
28	Kreekrak, Netherlands	Coastal-on dyke wall	3.65	(Musters et al. 1996)
29	Washington, USA	Grassland, deciduous riparian forest and scrub along drainages	3.21-3.59	(Erickson et al. 2003)
30	Alameda County, California, USA	Grassland	3.42	(Smallwood et al. 2018)
31	Tjaereborg, Denmark	Coastal grassland	3	(Pedersen & Poulsen 1991)
32	Island in Fehmarn, Germany	Flat island	3	(Welcker et al. 2017)
33	Blyth, Northumberland	Coastal shoreline	2.52	(Painter et al. 1999)
34	North-eastern Greece	Forests, sclerophyllous vegetation and alpine meadows and agricultural land	2.08	(Georgiakakis et al. 2012)
35	Buffalo Ridge, USA	Agricultural land and grassland	0.07-2.04	(Johnson et al. 2003)
36	Maharashtra, Satara	Rocky Plateau	1.9	(Pande et al. 2013)
37	Contra Costa County, California, USA	Grassland	1.82	(Smallwood et al. 2010)
38	Southern Great Plains, Oklahoma, USA	Mixed grasslands ecoregion	1.19–1.71	(Piorkowski & O'Connell 2010)
39	Osório, southern Brazil	Pasture for cattle and rice plantation, very close to a rain forest area	1.49	(Barros et al. 2015)
40	Blyth, Northumberland, UK	Coastal shoreline	1.34	(Still et al.1996)
41	Çanak-kale, İstanbul, and Hatay, Turkey	Mixed forest and partially cultivated areas	1.32	(Arikan & Turan 2017)
42	Southwestern Minnesota, USA	Upland prairie and prairie wetlands	0.33-0.66	(Osborn et al. 2000)
43	Harapanahalli, Davanagere, Karnataka	Dry forests	0.47	(Arun et al. 2015)
44	Jhangi, Gujarat	Marshy	0.38	(Kumar et al. 2012)
45	Navarra, Spain	Inland hills	0.34	(SEO/ BirdLife 1995)
46	Navarra, Spain	Inland hills	0.34	(Lekuona 2001)

47	England, Scotland and Wales		0.008-0.28	(Minderman et al. 2015)
48	Tarifa, Spain	Mountains, mosaic of forest, dense scrubland and pastures	0.27	(Barrios & Rodríguez 2004)
49	Chitradurga and Gadag, Karnataka, India	Southern tropical dry deciduous and thorn forest	0.26	Present study
50	Southern Alberta, Canada	Agricultural land	0.15-0.23	(Brown & Brenda 2006b)
51	San Cristóbal Island, Galapagos	Agricultural lands, near to National Park	0.21	(Cruz-Delgado et al. 2010)
52	California	Non-native annual grassland	0.2	(Thelander et al. 2003)
53	Italy	Low mountain scrubland and hemicryptophytic pasture patches	0.19	(Ferri et al. 2010)
54	Altamont, California	Ranchland	0.15	(Thelander & Rugge 2000)
55	Smøla, Norway	Sea coast	0.13	(Follestad et al. 2007)
56	Livermore, California, USA	Mixed oak woodland and savannah and open grassland	0.1	(Hunt & Hunt 2006)
57	Novar	Upland Moorland	0.08	(Bioscan 2001)
58	Altamont, California, USA	Ranchland	0.02-0.05	(Orloff & Flannery 1992)
59	California, USA		0.05	(Howell & DiDonato 1991)
60	Port Burwell, Ontario, Canada	Agricultural land	0.028-0.049	(Dance 2011)
61	Ovenden Moor, south Pennines	Upland Moorland	0.04	(EAS 1997)
62	Cemmaes, Wales	Upland Moorland	0.04	(Dulas 1995)
63	Tarifa, S. Spain	Coastal hills	0.03	(Janss 1998)
64	California, USA	--	0.03	(Howell 1997)
65	San Gorgonio	Barren to brittle bush	0.006	(Anderson et al. 2005)
66	Haverigg, Cumbria	Coastal grassland	0	(SGS Environment 1994)
67	Bryn Tytli, Wales	Upland Moorland	0	(Tyler 1995)
68	Utgrunden	Offshore	0	(Petterson & Stalin 2003)
69	Friedrich –Wilhelm-Lubke-Koog	Agricultural land	0	(Grünkorn et al. 2005)

References:

1. Winkelman, J. E. Vogels en het Windpark nabij Urk (NOP): Aanvaringsslachtoffers en Verstoring van Pleisterende Eenden, Ganzen en Zwanen. RIN-report 89/15. Arnhem (1989).

2. Kerns, J. & Kerlinger, P. A study of bird and bat collision fatalities at the Mountaineer Wind Energy Center, Tucker County, West Virginia: Annual report for 2003. Prepared for FPL Energy and Mountaineer Wind Energy Center Technical Review Committee (2004).
3. Cabrera-Cruz, S. A. *et al*. Estimates of aerial vertebrate mortality at wind farms in a bird migration corridor and bat diversity hotspot. *Glob*. *Ecol*. *Conserv*. **22**, p.e00966; https://doi.org/10.1016/j.gecco.2020.e00966 (2020).
4. Camina, Á. Bat fatalities at wind farms in northern Spain—lessons to be learned. *Acta*. *Chiropt*. **14**, 205-212 (2012).
5. Aschwanden, J. *et al*. Bird collisions at wind turbines in a mountainous area related to bird movement intensities measured by radar. *Biol*. *Conserv*. **220**, 228-236 (2018).
6. Krijgsveld, K. L., Akershoek, K., Schenk, F., Dijk, F. & Dirksen, S. Collision risk of birds with modern large wind turbines. *Ardea*. **97**, 357-366 (2009).
7. Grodsky, S. M., Jennelle, C. S., Drake, D. & Virzi, T. Bat mortality at a wind-energy facility in southeastern Wisconsin. *Wildl*. *Soc*. *Bull*. **36**, 773-783 (2012).
8. Everaert J., Devos K. & Kuijken E. Windturbines en vogels in Vlaanderen: voorlopige onderzoeksresultaten en buitenlandse bevindingen. Instituut voor Natuurbehoud, Rapport 2002.3, Brussel (2002).
9. Fiedler, J. K. Assessment of Bat Mortality and Activity at Buffalo Mountain Windfarm, Eastern Tennessee. MSc thesis, University of Tennessee (2004).
10. Everaert, J. Collision risk and micro-avoidance rates of birds with wind turbines in Flanders. *Bird Study*. **61**, 220-230 (2014).
11. Baerwald, E. F. & Barclay, R. M. Patterns of activity and fatality of migratory bats at a wind energy facility in Alberta, Canada. *J. Wildl*. *Manag*. **75**, 1103-1114 (2011).
12. Everaert, J. & Eric W. M. S. Impact of Wind Turbines on Birds in Zeebrugge (Belgium). *Bio*. *Conserv*. **16**, 3345–59 (2007).
13. Brown, W. K. & Brenda L. H. Monitoring of Bird and Bat Collisions with Wind Turbines at the Summer View Wind Power Project, Alberta 2005-2006. A report prepared for Vision Quest Wind electric, 42; https://tethys.pnnl.gov/sites/default/files/publications/BrownSummerfield-2006.pdf (2006a).
14. Doty, A. C. & Martin, A. P. Assessment of bat and avian mortality at a pilot wind turbine at Coega, Port Elizabeth, Eastern Cape, South Africa. *N. Z. J. Zool*. **40**, 75-80 (2013).

15. Zimmerling, J. R. & Francis, C. M. Bat mortality due to wind turbines in Canada. The *J. Wildl. Manag*. **80**, 1360-1369 (2016).
16. Winkelman, J. E. The impact of the SEP wind park near Oosterbierum (Fr.), on birds, 1: collision victims. RIN-report 92/2 DLO-Instutuut voor Bos- en Natuuronderzoek, Arnhem (1992).
17. Schaub, T., Klaassen, R. H., Bouten, W., Schlaich, A. E. & Koks, B. J. Collision risk of Montagu's Harriers Circus pygargus with wind turbines derived from high-resolution GPS tracking. *Ibis*. **162**, 520-534 (2020).
18. Grodsky, S. M., Jennelle, C. S. & Drake, D. Bird mortality at a wind-energy facility near a wetland of international importance. *Condor*. **115**, 700-711 (2013).
19. Zimmerling, J., Pomeroy, A., d'Entremont, M. & Francis, C. Canadian estimate of bird mortality due to collisions and direct habitat loss associated with wind turbine developments. *Avian Conserv*. *Ecol*. **8**, (2013).
20. Nicholson, C. P., Tankersley Jr, R. D., Fiedler, J. K. & Nicholas, N. S., Assessment and prediction of bird and bat mortality at wind energy facilities in the southeastern United States. Tennessee Valley Authority, Knoxville, Tennessee (2005).
21. Jain, A. A., Koford, R. R., Hancock, A. W. & Zenner, G. G. Bat mortality and activity at a northern Iowa wind resource area. The American midland naturalist, **165**, 185-200 (2011).
22. Crotty, M. L., Malek, A. L., Reding, D. M. & Roppe, J. Impacts of a single turbine wind facility on bat activity in Northeast Iowa http://www.nationalwind.org/wp-content/uploads/2014/04/23_Roppe.pdf (2014).
23. Onrubia A. *et al*. Estudio de la incidencia sobre la fauna -aves y quirópteros- del parque eólico de Elgea (Alava). Informe inédito de Consultora de Recursos Naturales, S.L. para Eólicas de Euskadi. Vitoria-Gasteiz (2002).
24. Johnson, G. D. *et al*. Collision mortality of local and migrant birds at a large-scale wind-power development on Buffalo Ridge, Minnesota. *Wildl*. *Soc*. *Bull*. 879-887 (2002).
25. Bull, L. S., Fuller, S. & Sim, D. Post-construction avian mortality monitoring at Project West Wind. *N. Z. J. Zool*. **40**, 28-46 (2013).
26. Choi, D. Y., Wittig, T. W. & Kluever, B. M. An evaluation of bird and bat mortality at wind turbines in the Northeastern United States. *PloS one*. **15**, p.e0238034; https://doi.org/10.1371/journal.pone.0238034 (2020).
27. Graff, B. J., Grovenburg, T. W., Jensen, K. C. & Jenks, J. A. Assessing Direct Impacts to Avifauna at Wind Energy Facilities in the Dakotas (2014).

28. Musters, C. J. M., Noordervliet, M. A. W. & Ter-Keurs, W. J. Bird casualties caused by a wind energy project in an estuary. *Bird Study*. **43**, 124–126 (1996).
29. Erickson, W. P., Kronner, K. & Gritski, B. Nine Canyon Wind Power Project avian and bat monitoring report. Nine Canyon Technical Advisory Committee (2003).
30. Smallwood, K. S. *et al*. Estimating wind turbine fatalities using integrated detection trials. *J. Wildl. Manag*. **82**, 1169-1184 (2018).
31. Pedersen, M. B. & Poulsen, E. Avian response to the implementation of the Tjaereborg wind turbine at the Danish Wadden Sea. Danske Vildtundersoegelser (Denmark) (1991).
32. Welcker, J., Liesenjohann, M., Blew, J., Nehls, G. & Grünkorn, T. Nocturnal migrants do not incur higher collision risk at wind turbines than diurnally active species. *Ibis*. **159**, 366-373 (2017).
33. Painter, A., Little, B. & Lawrence, S. Continuation of Bird Studies at Blyth Harbour Wind Farm and the Implications for Offshore Wind Farms. Report by Border Wind Limited DTI, ETSU W/13/00485/00/00 (1999).
34. Georgiakakis, P. *et al*. Bat fatalities at wind farms in north-eastern Greece. *Acta Chiropt.* **14**, 459-468 (2012).
35. Johnson, G. D. *et al.* Mortality of bats at a large-scale wind power development at Buffalo Ridge, Minnesota. *Am. Midl. Nat*. **150**, 332-342 (2003)
36. Pande, S. *et al*. CEPF Western Ghats Special Series: Avian collision threat assessment at Bhambarwadi Wind Farm Plateau in northern Western Ghats, India. Journal of Threatened Taxa, **5**, 3504-3515 (2013).
37. Smallwood, K. S., Bell, D. A., Snyder, S. A. & DiDonato, J. E. Novel scavenger removal trials increase wind turbine—caused avian fatality estimates. *J. Wildl. Manag*. 74, 1089-1096 (2010).
38. Piorkowski, M. D. & O'Connell, T. J. Spatial pattern of summer bat mortality from collisions with wind turbines in mixed-grass prairie. *Am. Midl. Nat*. **164**, 260-269 (2010).
39. Barros, M. A., de Magalhães, R. G. & Rui, A. M. Species composition and mortality of bats at the Osório Wind Farm, southern Brazil. *Stud. Neotrop. Fauna. Environ.* **50** 31-39 (2015).
40. Still, D., Little, B. & Lawrence, S. The effect of wind turbines on the bird population at Blyth Harbour (No. ETSU-W--13-00394/REP). Border Wind Ltd. **27** (1996).
41. Arikan, K. & Turan, S. L. Estimation of bird fatalities caused by wind turbines in Turkey. *Fresenius. Environ. Bull*. **26**, 6543-6550 (2017).

42. Osborn, R. G., Higgins, K. F., Usgaard, R. E., Dieter, C. D. & Neiger, R. D. Bird mortality associated with wind turbines at the Buffalo Ridge Wind Resource Area, Minnesota. *Am. Midl. Nat.* **143**, 41-52 (2000).
43. Arun, P. R., Jayapal, R. & Anoop, V. Impact of Hara wind power project of CLP wind farms (India) ltd. On wildlife including migratory birds and raptors at Harpanahalli, Davangere, Karnataka. Final report Submitted to CLP Windfarms (India) Pvt. Ltd. SACON Report. 145 (2015).
44. Kumar, S. R., Ali, A. & Arun, P. R. Impact of wind turbines on birds: a case study from Gujarat, India. *Sci. J. Environ. Sci.* **228**, 1-12 (2012).
45. SEO/BirdLife. Effects of wind power plants in the Campo de Gibraltar region. SGS Environment (1994). Haverigg Windfarm Ornithological Monitoring Programme. Report to Windcluster Ltd. (1995).
46. Lekuona, J. M. Uso del espacio por la avifauna y control de la mortalidad de aves y murciélagos en los parques eólicos de Navarra durante un ciclo anual. Direccion General de Medio Ambiente, Gobierno de Navarra, Pamplona (2001).
47. Minderman, J., Fuentes-Montemayor, E., Pearce-Higgins, J. W., Pendlebury, C. J. & Park, K. J. Estimates and correlates of bird and bat mortality at small wind turbine sites. *Biodivers. Conserv.* **24**, 467-482 (2015).
48. Barrios, L. & Rodriguez, A. Behavioural and environmental correlates of soaring-bird mortality at on-shore wind turbines. *J. Appl. Ecol.* **41**, 72-81 (2004).
49. Brown, W. K. & Brenda L. H. Bird and Bat Interactions with Wind Turbines, Castle River Wind Farm, Alberta. 33; https://tethys.pnnl.gov/sites/default/files/publications/Castle-River-Wind-Farm-Alberta.pdf (2006b).
50. Cruz-Delgado, F., Wiedenfeld, D. A. & González, J. A. Assessing the potential impact of wind turbines on the endangered Galapagos Petrel *Pterodroma phaeopygia* at San Cristóbal Island, Galapagos. *Biodivers. Conserv.* **19**, 679-694 (2010).
51. Thelander, C. G., Smallwood, K. S. & Rugge, L. Bird Risk Behaviors and Fatalities at the Altamont Pass Wind Resource Area. Subcontractor Report NREL/SR-500-33829 (December) https://256stuff.com/forums/attach/21/33829.pdf (2003).
52. Ferri, V., Locasciulli, O., Soccini, C. & Forlizzi, E. Post construction monitoring of wind farms: first records of direct impact on bats in Italy. *Hystrix.* **22** (2010).
53. Thelander, C. G. & Rugge, L. Avian Risk Behavior and Fatalities at the Altamont Wind Resource Area March 1998 to February 1999. Subcontractor Report NREL/SR-500-27545 (May) (2000).

54. Follestad, A., Flagstad, Ø., Nygård, T., Reitan, O. & Schulze, J. E. Vindkraft og fugl på Smøla 2003–2006. NINA rapport (2007).
55. Hunt, G. & Hunt, T. The trend of golden eagle territory occupancy in the vicinity of the Altamont Pass Wind Resource Area: 2005 survey. Unpublished report of the California Energy Commission, PIER Energy-Related Environmental Research, CEC-500-2006-056. URL www.energy.ca.gov/2006publications/CEC-500-2006-056/CEC-500-2006-056.pdf (2006).
56. Bioscan (UK) Ltd. Novar Windfarm Ltd Ornithological Monitoring Studies - Breeding bird and birdstrike monitoring 2001 results and 5-year review. Report to National Wind Power Ltd (2001).
57. Orloff, S. & Flannery, A. Wind Turbine Effects on Avian Activity, Habitat Use and Mortality in Alta- Mont Pass and Solano County Wind Resource Areas. Report to the Planning Departments of Alameda, Contra Costa, and Solano Counties and the California Energy Commission. (1992).
58. Howell, J. A. Bird mortality at rotor swept area equiva- lents, Altamont Pass and Montezuma Hills, California. *Transactions of the Western Section of the Wildlife Society* **33**, 24-29, (1997).
59. Dance, K. Raptor Mortality and Behavior at Wind Turbines Along the North Shore of Lake Erie During Autumn Migration 2006-2007. MSc thesis, University of Waterloo. https://uwspace.uwaterloo.ca/bitstream/handle/10012/5797/Dance_Kevin.pdf?sequence=1&isAllowed=y (2011).
60. EAS. Ovenden Moor Ornithological Monitoring. Report to Yorkshire Windpower. Keighley: Ecological Advisory Service (1997).
61. Dulas Engineering Ltd. The Mynyddy Cemmaes windfarm impact study. Ecological Impact - Final report. https://inis.iaea.org/search/search.aspx?orig_q=RN:27019308 (1995).
62. Janss, G. Bird behavior in and near a wind farm in Tarifa, Spain: management considerations. Avian Workgroup of the National Wind Coordinating Meeting, San Diego, Cal- ifornia, USA (1998).
63. Howell, J. A. Bird mortality at rotor swept area equiva- lents, Altamont Pass and Montezuma Hills, California. *Transactions of the Western Section of the Wildlife Society* **33**, 24-29 (1997).

64. Anderson, R. *et al.* Avian monitoring and risk assessment at the San Gorgonio Wind Resource Area (No. NREL/SR-500-38054). National Renewable Energy Lab.(NREL), Golden, CO (United States) https://doi.org/10.2172/15020049 (2005).
65. SGS Environment. Haverigg windfarm ornithological monitoring programme. Report to Windcluster Ltd. (1994).
66. Tyler, S. J. Bird strike study at Bryn Titli windfarm, Rhayader. Report to National Wind Power Ltd. (1995).
67. Petterson, J. & Stalin. T. Influence of offshore windmill on migratory birds in southeast coast of Sweden. Report to GE Wind Energy. Piorkowski (2003).
68. Grünkorn T., Diederichs A., Stahl B., Pöszig D. & Nehls G. Entwicklung einer Methode zur Abschätzung des Kollisionsrisikos von Vögeln an Windenergie-anlagen. Bioconsult SH, Hockensbüll, Germany. http//:www.umweltdaten.landsh.de/nuis/upool/gesamt/wea/voegel_ wea.pdf (2005).
